# Exploratory randomized open-label clinical trial of *Triphalā Kaṣāya* vaginal douche (*Yoni prakṣālana*) with and without *Gomūtra Arka* in vaginal candidiasis

**DOI:** 10.3389/fmed.2026.1910053

**Published:** 2026-08-13

**Authors:** Roshini Chandrashekar, Anjaly Muraleedharan, Hemavathi Shivapura Krishnarajabhatt, Cinu Mithra Nisa Sushilal, Parvathy Unnikrishnan

**Affiliations:** Department of Stri Roga and Prasuti Tantra (Gynecology and Obstetrics), Amrita School of Ayurveda, Amrita Vishwa Vidyapeetham, Amritapuri, Kollam, India

**Keywords:** ayurveda, cow urine distillate, image j software, triphala kashaya, vaginal candidiasis, Gomutra Arka

## Abstract

**Background:**

Vaginal candidiasis is the second most common cause of vaginal infection worldwide, affecting nearly 70–75% of women at least once during their lifetime, with recurrence in approximately 45%. In Ayurveda, its clinical presentation resembles *Ślaiṣmikī Yonivyāpad*. Preliminary clinical evidence suggests *Yoni Prakṣālana* (vaginal douche) with *Triphalā Kaṣāya* improves the clinical symptoms within the first 7 days of therapy. Classical Ayurvedic texts recommended combining *Triphalā Kaṣāya* and *Gomūtra* for *srava-pradhāna Ślaiṣmikī Yonivyāpat*, but, to date, no randomized controlled studies have explored the potential additive effect of *Gomūtra Arka* with *Triphalā Kaṣāya* using microbiological outcome assessment.

**Objective:**

To generate preliminary clinical and microbiological outcome estimates and evaluate the safety of adding *Gomūtra Arka* to *Triphalā Kaṣāya Yoni Prakṣālana* in women with vaginal candidiasis, thereby informing the design of future confirmatory randomized controlled trials.

**Methods:**

A single-center, open-label, randomized exploratory clinical trial (CTRI/2024/08/072177) enrolled 36 of 42 screened participants and randomized (1:1 allocation) into two groups (18 per group). 32 participants (16 per group) completed study and were analyzed. Group A received *Triphalā Kaṣāya Yoni Prakṣālana* alone, and Group B received *Triphalā Kaṣāya* combined with *Gomūtra Arka* for 7 consecutive days. Outcomes were assessed on Day 0, 3, 5, and 8. The exploratory primary outcome was change in candida spore count quantified using ImageJ software. Exploratory secondary outcome was changes in vaginal discharge and pruritus, assessed using discharge grading scale and the 5-D Pruritus Scale. Safety was prospectively monitored throughout the intervention by adverse event surveillance.

**Results:**

Both groups showed improvement in clinical symptoms and microbiological parameters. Exploratory analyses suggested earlier improvements in vaginal discharge, pruritus and Candida spore count in the combination group. Exploratory analyses suggested between-group differences from Day 5 onwards; however, these observations should be interpreted as hypothesis-generating rather than confirmatory evidence.

**Conclusion:**

The addition of *Gomūtra Arka* to *Triphalā Kaṣāya Yoni Prakṣālana* was feasible and associated with favorable exploratory clinical and microbiological outcomes. These preliminary findings support adequately powered multicentre randomized controlled trials to confirm therapeutic effectiveness and further evaluate safety and mechanisms of action.

**Clinical trial registration:**

https://ctri.nic.in/Clinicaltrials/pmaindet2.php?EncHid=MTEwODU2&Enc=&userName=, identifier: CTRI/2024/08/072177.

## 1 Introduction

Women's health plays a crucial role in the well-being of families and communities, with reproductive health constituting an essential component across all stages of life, from menarche to menopause (1). Vaginal infections are among the most common gynecological conditions affecting women of reproductive age and are a frequent cause of healthcare consultation worldwide (2). Among these, vaginal candidiasis is the second most common vaginal infection, predominantly caused by *Candida albicans*, which accounts for nearly 85–90% of cases (3).

Globally, approximately 70–75% of women experience vaginal candidiasis at least once during their lifetime, while 5–9% develop recurrent vulvovaginal candidiasis (RVVC), defined as four or more symptomatic episodes annually (4). A Retrospective study by Rajalakshmi et al. conducted in Chennai, South India, found that vaginal candidiasis was the second most prevalent vaginal infection among women of reproductive age, following bacterial vaginosis (5). Tropical climatic conditions, including high humidity, damp environments, and prolonged moisture exposure, further predispose women to fungal infections (6). Clinically, vaginal candidiasis is characterized by intense pruritus, thick curdy white vaginal discharge, burning sensation, dyspareunia, and dysuria, all of which significantly impair quality of life (7).

The vaginal microbiota plays a vital role in maintaining vaginal homeostasis and is influenced by hormonal fluctuations, menstruation, pregnancy, sexual practices, and contraceptive use (8, 9). Disruption of this microbial balance predisposes to pathogen overgrowth and increases susceptibility to vaginal infections (10). If left untreated or recurrent, vaginal candidiasis may contribute to adverse reproductive and obstetric outcomes (11). Current management primarily includes oral and topical antifungal agents (12). However, increasing antifungal resistance, recurrent infections, treatment-associated adverse effects, and rising global disease burden remain major concerns. The annual global prevalence of recurrent vulvovaginal candidiasis is estimated to be 138–150 million cases and is projected to increase further by 2030 (13). These challenges highlight the need for safe, cost-effective, and evidence-based complementary therapeutic approaches.

In Ayurveda, diseases affecting the female reproductive system are described under *Yonivyāpat*. Among them, *Ślaiṣmikī Yonivyāpat* is characterized by *picchila (sliminess), śitala srava (secretions)* from *yoni (vagina)* associated with *kandu (itching), alpa-vedanā (mild pain)*, or *avedanā (painless)* (14), which closely resemble the clinical manifestations of vaginal candidiasis. Ayurveda recommends ṛ*ukṣa* (dry) and *uṣṇa cikitsā (hot)* for the management of *kapha*- and *kleda*-dominant conditions such as *Ślaiṣmikī Yonivyāpat* (15). Several *sthānika cikitsā* (local treatment) procedures have been described for *Yonivyāpat*, among which *Yoni Prakṣālana* (vaginal douche) is considered an important local therapeutic intervention.

Classical Ayurvedic texts, including Charaka Samhita and Aṣṭāṅga Saṅgraha, mention *srava (excessive vaginal discharge)* as a cardinal feature of *Ślaiṣmikī Yonivyāpat*. In this context, the combined use of *Triphalā Kaṣāya* along with *Gomūtra* is recommended as therapeutic agents owing to their kaphahara, shodhana and antimicrobial properties, providing a traditional rationale for their evaluation in vaginal candidiasis (16). Experimental evidence has demonstrated antifungal activity of cow urine distillate (*Gomūtra Arka*/Cow Urine Distillate) against *Candida* species, supporting its potential therapeutic application in fungal infections (17). As a distilled preparation, *Gomūtra Arka* is considered comparatively safe, economical, with a longer shelf life (1 year) and suitable for local therapeutic use.

Prior to the present clinical study, an *in vitro* analytical evaluation was conducted to determine the minimum inhibitory concentration (MIC) and optimize the combination ratio of *Triphalā Kaṣāya* and *Gomūtra Arka* against Candida species. The ratio of 4:1 (here 4 parts of *Triphalā Kaṣāya*, 1 part of *Gomūtra Arka* was added, i.e. 800:200) demonstrated the highest antifungal efficacy and was therefore selected as the standardized intervention ratio for the present clinical trial; the in-vitro study reports are attached as Supplementary material to this study. Clinical outcomes in vaginal candidiasis can be assessed using validated symptom scales such as the 5-D Pruritus Scale (18) and discharge grading scale (19), while fungal spore quantification using KOH wet mount microscopy with ImageJ software analysis enables objective microbiological assessment (20, 21).

*Triphalā Kaṣāya* was selected as the comparator because it is a textually indicated and routinely practiced local Ayurvedic intervention for *srava-pradhāna yonivyāpad*, including conditions clinically comparable to vaginal candidiasis. A preliminary clinical study conducted at Amrita School of Ayurveda, Kerala, demonstrated that *Yoni Prakṣālana* (vaginal douche) using *Triphalā Kaṣāya* was associated with significant improvement in the clinical manifestations of vaginal candidiasis following 10 days of intervention. However, clinically significant symptomatic improvement was observed within the first seven days of therapy, indicating that clinical improvement could be achieved during this period, and scope for improving the rate of therapeutic response. Based on classical Ayurvedic recommendations advocating the combined use of Triphalā Kaṣāya and Gomutra, together with the findings from *in vitro* analytical study, we hypothesized that the addition of Gomutra Arka could enhance the therapeutic efficacy of *Triphalā Kaṣāya* by accelerating fungal clearance and symptom resolution. Accordingly, the present study was designed to evaluate the incremental therapeutic contribution of Gomutra Arka over an already established Ayurvedic local intervention, rather than to compare Ayurvedic therapy with conventional antifungal treatment. Therefore, a 7-day intervention schedule was selected for the present study to evaluate the therapeutic response while potentially enhancing treatment adherence (22). However, no randomized controlled trials to date have evaluated the combined use of *Triphalā Kaṣāya* and *Gomūtra Arka* with objective microbiological assessment of fungal spore clearance and accelerated symptom resolution.

The present exploratory randomized clinical trial was designed to generate preliminary clinical and microbiological outcome estimates of adding *Gomūtra Arka* to *Triphalā Kaṣāya Yoni Prakṣālana* in the management of vaginal candidiasis (*Ślaiṣmikī Yonivyāpat*)while evaluating intervention safety, thereby informing the design of future adequately powered confirmatory randomized controlled trials.

### 1.1 Exploratory primary objective

To explore changes in *Candida* spore count assessed by KOH wet mount microscopy from baseline to Day 3, Day 5, and Day 8 following intervention with *Triphalā Kaṣāya Yoni Prakṣālana* with *Gomūtra Arka* vs. *Triphalā Kaṣāya Yoni Prakṣālana* alone.

### 1.2 Exploratory secondary objectives

- To explore changes in 5-D Pruritus Scale scores from baseline to Day 3, Day 5, and Day 8 between the two intervention groups.
- To explore changes in vaginal discharge grading scores from baseline to Day 3, Day 5, and Day 8 between the two intervention groups.

## 2 Materials and methods

### 2.1 Patient and public involvement

Patients and members of the public were not involved in the design, conduct, reporting, or dissemination planning of this study.

Participation was entirely voluntary, and written informed consent was obtained from all participants before enrolment and prior to the commencement of any study-related procedures.

### 2.2 Trial design and ethical considerations

This study was designed as a single-center, open-label, parallel-group exploratory randomized controlled clinical trial with a 1:1 allocation ratio. The exploratory design was adopted to prospectively evaluate the short-term safety of the intervention and generate preliminary clinical and microbiological outcome estimates associated with the addition of *Gomūtra Arka* to *Triphalā Kaṣāya Yoni Prakṣālana* compared with *Triphalā Kaṣāya Yoni Prakṣālana* alone. The comparator was intentionally selected as *Triphalā Kaṣāya* alone to isolate the incremental therapeutic contribution of *Gomūtra Arka* over an active ayurvedic intervention rather than to establish direct comparison with conventional antifungal therapy.

An exploratory framework was selected because no prior randomized controlled clinical trials were available evaluating this combination intervention with objective microbiological outcome assessment in vaginal candidiasis. Therefore, the present study was intended to generate preliminary estimates that may inform the design of future adequately powered confirmatory randomized controlled trials rather than to establish definitive evidence of therapeutic efficacy.

The study protocol was approved by the Institutional Ethics Committee (IEC) of Amrita School of Ayurveda, Kerala, India. All study procedures were conducted in accordance with the ethical principles of the Declaration of Helsinki and applicable national guidelines for biomedical research involving human participants. The study was prospectively registered with the Clinical Trial Registry of India (CTRI/2024/08/072177).

The corresponding author is a member of the Institutional Ethics Committee of Amrita School of Ayurveda. However, the corresponding author was not involved in the ethical review, discussion, decision-making, or approval process of this study protocol in order to avoid any potential conflict of interest.

#### 2.2.1 Changes to trial protocol

No major modifications were made to the study methodology, eligibility criteria, interventions, outcome measures, or statistical analysis plan after trial commencement. All reported analyses and outcomes were prespecified in the registered study protocol. The reporting of this exploratory randomized clinical trial was prepared in accordance with the CONSORT 2025 Statement for randomized trials, and the intervention was described following the principles of the TIDieR (Template for Intervention Description and Replication) checklist to enhance methodological transparency and facilitate independent replication of the study.

### 2.3 Trial setting

The study was conducted at the Department of Prasuti Tantra and Strīroga, Amrita School of Ayurveda, Amritapuri, Kollam, Kerala, India, a teaching hospital and research center under Amrita Vishwa Vidyapeetham. Participant recruitment, eligibility screening, informed consent, intervention delivery, clinical outcome assessment, specimen collection, and follow-up evaluations were performed according to a prespecified study protocol. All intervention procedures were carried out in a dedicated procedure theater under strict aseptic precautions using a standardized operating procedure (SOP) to ensure procedural consistency, participant safety, and reproducibility throughout the study.

### 2.4 Eligibility criteria

Written informed consent was obtained from all eligible participants before enrolment and prior to the commencement of any study-related procedures following a detailed explanation of the study objectives, interventions, potential benefits, possible risks, and participants' rights, including the right to withdraw from the study at any stage without affecting their standard medical care.

#### 2.4.1 Inclusion criteria

Participants fulfilling all of the following criteria were included:

- Women aged 18–45 years
- Presence of clinical symptoms suggestive of vaginal candidiasis
- Demonstration of fungal hyphae/spores in KOH wet mount microscopy, confirming candidial infection.

The age range was selected to include women in the reproductive age group, in whom vaginal candidiasis is most commonly encountered.

#### 2.4.2 Exclusion criteria

Participants were excluded if any of the following conditions were present:

- Pregnancy
- Diabetes mellitus
- Sexually transmitted diseases or pelvic inflammatory disease
- Known genital or systemic malignancies
- Current oral contraceptive pill use
- Current systemic antibiotic therapy.

These exclusion criteria were selected to minimize confounding factors that could independently alter vaginal microbiota, influence candida growth, interfere with symptom assessment, or affect treatment response.

#### 2.4.3 Personnel delivering the intervention

All *Yoni Prakṣālana* interventions were administered by the Principal Investigator from the Department of Prasuti Tantra and Strīroga, who was trained in the standardized study procedure. Conducting all intervention procedures by a single trained investigator helped maintain procedural consistency and uniformity and minimized operator-dependent variation throughout the study.

All the *Yoni Prakṣālana* procedures were administered by the Principal Investigator from the Department of Prasuti Tantra and Strīroga, who received standardized training in the study intervention before trial initiation. All interventions were delivered according to a predefined Standard Operating Procedure (SOP), thereby ensuring uniformity in intervention preparation, administration technique, treatment duration, participant positioning, and procedural execution throughout the study. Administration of all interventions by a single trained investigator minimized operator-dependent variation and ensured procedural consistency.

### 2.5 Study participants and recruitment

Women presenting with symptoms suggestive of vaginal candidiasis, including vulvovaginal pruritus, abnormal vaginal discharge, burning sensation, dyspareunia, and vulvovaginal discomfort, were consecutively screened for eligibility at the Outpatient and Inpatient Departments of the Department of Prasuti Tantra and Strīroga, Amrita School of Ayurveda, Kerala, India. Eligibility assessment, informed consent, detailed clinical history, physical examination, and participant enrolment were undertaken by the Principal Investigator according to the predefined study protocol under the supervision of the study investigators.

Vaginal specimens were collected by the Principal Investigator under strict aseptic precautions using a sterile vaginal swab. The specimens were immediately transported to the Department of Microbiology, where KOH wet mount microscopy was independently performed by a qualified microbiologist according to the institutional laboratory standard operating procedure. Vaginal candidiasis was microbiologically confirmed by the demonstration of fungal hyphae and/or blastospores on KOH wet mount examination (21). Although fungal culture remains the reference standard for species identification, KOH microscopy was selected because of its rapid turnaround time, feasibility, and suitability for repeated short-interval microbiological assessment in the present exploratory trial.

Following microbiological confirmation and written informed consent, 36 eligible participants were randomly allocated in a 1:1 ratio to receive either *Triphalā Kaṣāya Yoni Prakṣālana* alone (control group) or *Triphalā Kaṣāya Yoni Prakṣālana* combined with *Gomūtra Arka* (intervention group), with 18 participants assigned to each treatment arm.

All participants received the allocated intervention according to the predefined study protocol. Intervention procedures were performed by the Principal Investigator in a dedicated procedure theater under strict aseptic precautions following the standardized SOP. Clinical assessments and vaginal specimen collection were undertaken at baseline (Day 0) and during follow-up on Days 3, 5, and 8. Subjective clinical outcomes were evaluated by an independent assessor who was not involved in intervention administration, while microbiological assessments were independently performed by a microbiologist. Candida spore quantification was subsequently carried out using ImageJ-assisted image analysis according to the predefined analysis protocol.

**Figure 1:**
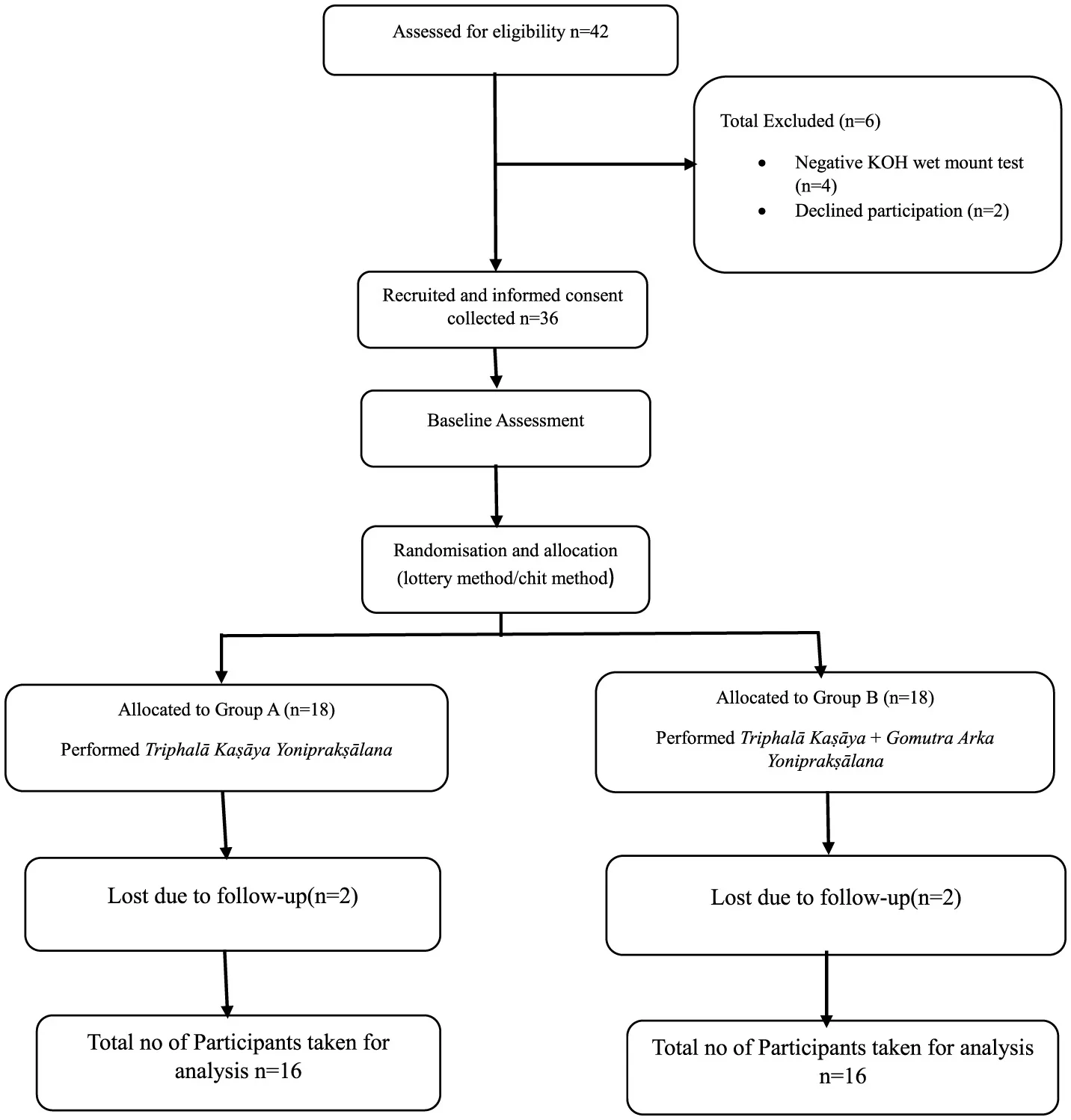
CONSORT flow diagram of participant recruitment, randomization, follow-up, and analysis.

A total of 42 women were screened for eligibility. Four participants were excluded because KOH wet mount microscopy was negative for Candida, and two declined participation. Thirty-six eligible participants were randomized. During the intervention period, two participants from each group discontinued treatment for personal reasons unrelated to the intervention. All participants were prospectively monitored for adverse events throughout the study using the predefined safety monitoring protocol, and no intervention-related serious adverse events or clinically significant safety concerns were observed. Consequently, 32 participants (16 per group) completed the study and were included in the per-protocol analysis. The participant flow is illustrated in the CONSORT diagram (Figure 1).

Recruitment, intervention delivery, follow-up, and outcome assessment were conducted between August 2024 and November 2025.

#### 2.5.1 Sample size

The present study was designed as an exploratory randomized controlled clinical trial intended to generate preliminary estimates of clinical and microbiological outcomes rather than establish confirmatory therapeutic efficacy. Accordingly, the sample size estimation was performed to provide a rational and transparent basis for participant recruitment and preliminary effect estimation for future adequately powered confirmatory randomized controlled trials, rather than as a formal confirmatory power calculation.

Although formal hypothesis-driven sample size calculations are not mandatory for exploratory clinical trials, a conventional two-sample comparison of means was used to estimate a feasible sample size. The calculation was based on the exploratory primary microbiological outcome, namely Candida spore count assessed by KOH wet mount microscopy and quantified using ImageJ-assisted analysis. The anticipated mean difference and pooled variance were derived from pilot estimates obtained from a preliminary clinical study conducted at Amrita School of Ayurveda in women with vaginal candidiasis who received *Triphalā Kaṣāya Yoni Prakṣālana*, thereby providing estimates that were directly relevant to the present study population and intervention.

The sample size was estimated using the following formula for comparison of two independent means:


$$\begin{array}{l} n=\frac{2s_{p}^{2}{\left[Z_{\left(1-\alpha /2\right)}+Z_{\left(1-\beta \right)}\right]}^{2}}{\mu_{d}^{2}} \end{array}$$

Where:

- $s_{p}^{2}=$ pooled variance
- $s_{1}^{2}=$ variance in Group A
- $s_{2}^{2}=$ variance in Group B
- μ_*d*_= anticipated mean difference between groups
- α= significance level
- 1 − β= nominal statistical power.

Based on the pilot estimates from the institutional study, the pooled variance ($s_{p}^{2}$) was 1.69, and the anticipated mean difference (μ_*d*_) was 1.29. Assuming a two-sided significance level of 0.05 and a nominal statistical power of 90%, the estimated sample size was 15.65 participants per group. After allowing for an anticipated attrition rate of 10%, the required sample size was rounded to 18 participants per group, resulting in a total sample size of 36 participants.

As the present investigation was exploratory in nature, this sample size estimation was intended to provide a rational basis for participant recruitment and preliminary estimation of treatment effects and should not be interpreted as a formal confirmatory power calculation.

### 2.6 Interventions and comparator

#### 2.6.1 Overview

All participants underwent *Yoni Prakṣālana* once daily for seven consecutive days according to a predefined Standard Operating Procedure (SOP). The intervention was administered by the Principal Investigator in the designated procedure theater of the Department of Prasuti Tantra and Strīroga, Amrita School of Ayurveda Hospital, under strict aseptic precautions.

Participants allocated to the intervention group received *Yoni Prakṣālana* using a formulation consisting of *Triphalā Kaṣāya* and *Gomūtra Arka* in a standardized ratio of 800 mL:200 mL (4:1), whereas participants in the control group received 1,000 mL of *Triphalā Kaṣāya* alone. Apart from the intervention formulation, both groups underwent identical treatment schedules, intervention procedures, follow-up visits, outcome assessments, and safety monitoring according to the predefined study protocol. This design enabled evaluation of the additional contribution of *Gomūtra Arka* while maintaining *Triphalā Kaṣāya* as the common base intervention.

#### 2.6.2 Trial drugs

*Triphalā Kaṣāya Curṇa* and *Gomūtra Arka* were procured from Amrita School of Ayurveda, Kerala, India, and Sri Sri Tattva, Bengaluru, India, respectively. Both formulations were manufactured under Good Manufacturing Practice (GMP) standards. The formulations were stored in airtight containers under standardized storage conditions until use to preserve their quality and stability throughout the study period.

#### 2.6.3 Intervention group

Participants allocated to the intervention group received *Yoni Prakṣālana* using a formulation consisting of 800 mL of freshly prepared *Triphalā Kaṣāya* combined with 200 mL of *Gomūtra Arka*. The intervention was administered once daily for seven consecutive days according to the predefined Standard Operating Procedure.

#### 2.6.4 Rationale for dose standardization

The intervention ratio (800 ml of *Triphalā Kaṣāya*: 200 ml *Gomūtra Arka)* was selected based on a prior *in vitro* antimicrobial activity study conducted at the Amrita Center for Advanced Research in Ayurveda, Kerala, India. Multiple ratios of *Triphalā Kaṣāya* and *Gomūtra Arka* were evaluated against Candida albicans using minimum inhibitory concentration (MIC) analysis to identify the ratio demonstrating maximum antifungal activity. The corresponding laboratory reports are provided as Supplementary material.

Among the formulations evaluated, the 4:1 (800:200) ratio demonstrated the highest inhibitory activity against Candida albicans and was therefore selected for clinical application in the present study. Incorporation of a pre-standardized ratio was intended to improve intervention reproducibility and methodological consistency.

#### 2.6.5 Method of preparation of Triphalā Kaṣāya

*Triphalā Kaṣāya* was freshly prepared each day by the Principal Investigator in the Department of Prasuti Tantra and Strīroga according to the classical method described in the Suśruta Saṃhitā. Briefly, 250 g of coarsely powdered *Triphalā Curṇa* was boiled with sixteen parts of water (4,000 mL) and reduced to one-fourth of the original volume (1,000 mL) (23). The decoction was subsequently filtered and administered in a lukewarm state. This method of preparation was selected based on findings from a preliminary *in vitro* study conducted at the Amrita Center for Advanced Research in Ayurveda, which demonstrated enhanced antimicrobial activity compared with alternative preparation methods (24).

#### 2.6.6 Standard operating procedure for Yoni Prakṣālana

Before each intervention, participants were advised to empty their bladder and were positioned in the dorsal recumbent position with flexed knees. Following routine perineal cleansing under strict aseptic precautions, the freshly prepared intervention formulation was transferred into a sterile douche can fitted with sterile tubing and a vaginal nozzle.

The nozzle was gently introduced into the vaginal canal, and the medicated formulation was allowed to flow uniformly to ensure adequate contact with the vaginal walls and fornices. The formulation was administered in a lukewarm state to improve participant comfort and procedural tolerability. After completion of the procedure, participants remained in the supine position for approximately 10 min before resuming normal activities. The intervention was administered once daily for seven consecutive days according to the predefined Standard Operating Procedure by the same trained investigator throughout the study to ensure procedural standardization and minimize operator-dependent variation.

#### 2.6.7 Monitoring and assessments

Clinical and microbiological assessments were performed at baseline (Day 0) and repeated on Days 3, 5, and 8 according to the predefined study schedule. Vaginal discharge grading and the 5-D Pruritus Scale were assessed by an independent clinical assessor who was not involved in intervention delivery. Vaginal specimens for KOH wet mount microscopy were collected by the Principal Investigator and analyzed independently by the Department of Microbiology according to the institutional laboratory standard operating procedure. Candida spore quantification was subsequently performed using ImageJ-assisted image analysis.

Participants were prospectively monitored for adverse events throughout the intervention period, and treatment adherence was documented using daily treatment records. The repeated assessment schedule was selected to characterize the temporal pattern of early clinical response and microbiological changes following the intervention.

### 2.7 Exploratory outcome measures

Consistent with the exploratory design of the present trial, all outcome measures were prespecified to generate preliminary clinical and microbiological data that may inform the design, outcome selection, and sample size estimation of future adequately powered confirmatory randomized controlled trials.

The exploratory primary outcome measure was the change in Candida spore count assessed by KOH wet mount microscopy and quantified using ImageJ-assisted image analysis. ImageJ-assisted quantification was incorporated to improve measurement objectivity and reduce observer-dependent variability during microscopic assessment of fungal burden.

The exploratory secondary outcome measures were:

- Change in pruritus severity assessed using the validated 5-D Pruritus Scale.
- Change in vaginal discharge severity assessed using a standardized vaginal discharge grading scale.

All outcome assessments were performed at baseline (Day 0) and repeated on Days 3, 5, and 8 according to the predefined assessment schedule.

### 2.8 Harms

Participants were prospectively monitored for adverse events throughout the study period by the Principal Investigator using the Naranjo Adverse Drug Reaction Probability Scale according to the predefined safety monitoring protocol. At each follow-up visit, participants were specifically evaluated for any local irritation, discomfort, allergic manifestations, or systemic adverse reactions potentially associated with the intervention.

No intervention-related serious adverse events, clinically significant safety concerns, or treatment discontinuations attributable to adverse reactions were observed during the study period.

### 2.9 Interim analysis and stopping guidelines

No interim analysis was planned or conducted because of the short intervention duration and exploratory nature of the study. Likewise, no formal stopping guidelines were established because the interventions were considered low risk and the treatment period was brief.

### 2.10 Randomization

#### 2.10.1 Sequence generation

Eligible participants were randomly allocated to the intervention or control group in a 1:1 ratio using a simple lottery-based randomization method. Sequentially numbered chits (1–36) were prepared, folded identically, and placed in an opaque container. Each participant independently selected one chit without replacement. Participants selecting odd-numbered chits were allocated to one treatment group, whereas those selecting even-numbered chits were allocated to the other group. This simple randomization procedure was considered appropriate for the relatively small sample size and exploratory nature of the present trial while ensuring equal probability of allocation.

#### 2.10.2 Allocation concealment

Allocation concealment was maintained until the moment of assignment by placing all sequentially numbered chits within an opaque container before participant recruitment commenced. Because group allocation remained unknown until a participant selected a chit, the procedure minimized selection predictability before allocation.

#### 2.10.3 Implementation

Participant recruitment, eligibility assessment, informed consent, randomization, intervention delivery, specimen collection, and follow-up assessments were coordinated by the Principal Investigator according to the predefined study protocol. Statistical analysis was performed independently by the biostatistician using the prespecified statistical analysis plan after completion of data collection.

#### 2.10.4 Blinding

Blinding of participants and the treating investigator was not feasible because of the nature of the intervention procedures and the characteristic odor of *Gomūtra Arka*. However, several measures were implemented to minimize assessment bias. Subjective clinical outcomes, including pruritus severity and vaginal discharge grading, were assessed by an independent assessor who was not involved in intervention delivery and remained unaware of treatment allocation. Vaginal swab specimens were collected by the Principal Investigator and independently examined by a qualified microbiologist who had no role in participant recruitment, treatment administration, or clinical outcome assessment. Candida spore quantification was subsequently performed using ImageJ-assisted image analysis according to a predefined analysis protocol to improve measurement objectivity and reduce observer-dependent variability. Although complete blinding was not feasible, these measures were implemented to minimize assessment bias within the methodological constraints of the exploratory trial design.

### 2.11 Statistical analysis

Data were entered into a predesigned Case Record Form (CRF) by the Principal Investigator. Following verification for completeness and accuracy, the final dataset was analyzed using Jamovi software (version 2.6.26) by an independent biostatistician according to a prespecified statistical analysis plan.

Baseline demographic and clinical characteristics were summarized separately for each study group. Continuous variables were expressed as mean ± standard deviation (SD) for normally distributed data and as median with interquartile range (IQR) for non-normally distributed data. Categorical variables were summarized as frequencies and percentages. Baseline comparisons between groups were undertaken to characterize the study population and assess baseline comparability rather than to establish statistical equivalence.

For non-normally distributed variables, within-group changes across repeated assessment time points were analyzed using the Friedman test followed by Durbin–Conover *post-hoc* comparisons, while between-group comparisons were performed using the Mann–Whitney *U* test. For normally distributed variables, within-group changes over time were analyzed using repeated-measures analysis of variance (ANOVA) followed by Tukey's *post-hoc* test, whereas between-group comparisons at individual assessment time points were performed using the independent-samples *t*-test.

The primary analysis followed a per-protocol approach. Participants who discontinued the intervention during the treatment period were excluded from the final analysis because repeated outcome measurements required for longitudinal analyses were incomplete.

As this was an exploratory randomized clinical trial, all statistical analyses were prespecified as exploratory and intended to characterize observed data patterns and generate preliminary estimates of treatment response. Accordingly, *p*-values, 95% confidence intervals, and effect size estimates are presented to facilitate interpretation of the observed data and should not be interpreted as confirmatory evidence of therapeutic efficacy or effectiveness. A two-sided significance level of 0.05 was used solely as a descriptive threshold for the exploratory analyses.

### 2.12 Withdrawal criteria

Participants were withdrawn from the study if they discontinued the intervention schedule, failed to attend follow-up assessments, or voluntarily withdrew consent during the study period. A total of four participants were withdrawn during the course of the study.

### 2.13 Patient education

Women were told to abstain from sexual intercourse until treatment is completed and symptoms have resolved, as it is sexually transmissible and to maintain local genital hygiene. They were advised to avoid tight-fitting synthetic clothing, local irritants such as perfumed products, soap gels and other vaginal wash solutions.

## 3 Results

### 3.1 Intervention and comparator delivery

All randomized participants had microbiological confirmation of *Candida* elements on KOH wet mount at baseline and received the allocated intervention according to the prespecified study protocol.

Participants allocated to the control group underwent *Triphalā Kaṣāya Yoni Prakṣālana* once daily for 7 consecutive days. Participants allocated to the intervention group underwent *Yoni Prakṣālana* using a standardized combination of *Triphalā Kaṣāya* and *Gomūtra Arka* once daily for 7 consecutive days.

All intervention procedures were administered by the Principal Investigator from the Department of Prasuti Tantra and Strīroga using the predefined standardized operating procedure to ensure procedural consistency and intervention fidelity throughout the study.

Treatment adherence was monitored using daily treatment records. Of the 36 randomized participants, 32 completed the prescribed 7-day intervention schedule without major protocol deviations. Overall, both interventions were implemented according to the study protocol, demonstrating good treatment adherence and procedural feasibility throughout the study period.

### 3.2 Concomitant care

Participants in both groups were instructed to avoid the use of systemic or topical antifungal medications, vaginal medications, probiotics, or any additional therapies that could potentially influence the study outcomes during the intervention period. No participant reported receiving concomitant treatment for vaginal candidiasis during the study period.

### 3.3 Baseline data

Baseline demographic and clinical characteristics were generally comparable between the two study groups (Table 1). No notable between-group differences were observed in age, duration of symptoms, baseline *Candida* spore count, or 5-D Pruritus score. However, participants in the intervention group presented with a higher baseline vaginal discharge grade than those in the control group. As this study employed simple randomization with a relatively small sample size, this baseline imbalance was considered during interpretation of the exploratory findings. No *post-hoc* statistical adjustment was performed because the study was designed as an exploratory randomized clinical trial.

**Table 1:** Baseline demographic and clinical characteristics for each group.

Baseline variable	Group A (*n* = 16)	Group B (*n* = 16)	*P*-value
Age (years)	31.38 ± 5.93	32.94 ± 6.30	0.476
Education	UG-10 (62.5%) PG-5 (31.2%) SSLC-1 (6.2%)	UG-11 (68.8%) PG-3 (18.8%) SSLC-2 (12.5.1%)	0.644
Occupation	Housewife- 8 (50%) Employed-8 (50%)	Housewife-11 (68.8%) Employed- 5 (31.5.5)	0.400
Nature of work	Hardworking- 9 (56.2%) Sedentary- 7 (43.8%)	Hardworking- 11 (68.7%) Sedentary- 5 (31.3%)	0.500
Religion	Hindu-9 (56.3%) Christian-3 (18.8%) Muslim-4 (25%)	Hindu-7 (43.8%) Christian- 5 (31.3%) Muslim- 4 (25%)	0.687
Domicile	Rural-9 (56.3%) Urban-7 (43.8%)	Rural- 5 (31.3%) Urban- 11 (68.8%)	0.285
Marital status	Married-16 (100%)	Married-16 (100%)	1.000
Economic status	Lower class- 1 (6.3%) Middle class- 14 (87.5%) Upper class- 1 (6.3%)	Lower class- 0 Middle class- 16 (93.8%) Upper class- 1 (6.3%)	0.245
BMI(Kg/m2)	24 ± 2.42	23.93 ± 2.48	0.753
Aggravation of symptoms (Day/Night)	Day & Night- 6 (37.5%) Night- 10 (62.5%)	Day & Night-7 (43.8%) Night- 9 (56.2%)	0.68
Sleep	Disturbed- 14 (87.5%) Sound- 2 (12.6%)	Disturbed- 13 (81.3%) Sound- 3 (18.8%)	0.63
Working atmosphere	Humid- 14 (87.6%) Cold- 2 (12.5%)	Humid- 16 (100%)	0.339
Sexually active	Active-10 (62.5%) Not active-6 (37.5%)	Active- 13 (81.3%) Not active- 3 (18.85)	0.432
Method adopted for genital cleaning	Soap water-12 (75%) V-Wash- 4 (25%) Nill-0	Soap water-11 (68.8%) V-Wash- 4 (25%) Nill-1 (6.3%)	0.594
Method adopted for the removal of Pubic hair	Shave-16 (100%)	Shave-16 (100%)	1.00
Prakruti	Vata Kapha- 13 (81.2%)	Vata Kapha- 13 (81.2%)	0.828
	Vata Pitta- 3 (18.8%)	Vata Pitta- 3 (18.8%)	
Baseline P/V discharge score (BT-D0)	G1-4 G2-9 G3-2	G1-0 G2-7 G3-9	0.011
Baseline 5-D pruritus score (BT-D0)	17.56 ± 4.86	16.81 ± 4.52	0.64
Baseline candida spore count (BT-D0)	408.12 ± 290.60	442.12 ± 246.1	0.724

Baseline comparability reveals that there was no statistically significant difference (*p* > 0.05) between group A and group B.

### 3.4 Numbers analyzed, outcomes and estimation

A total of 36 participants were randomized, with 18 participants assigned to each group. Thirty-two participants completed the intervention and all prespecified follow-up assessments through Day 8 and were included in the per-protocol analysis. Four participants (two from each group) discontinued the intervention for personal reasons before study completion and were excluded from the analysis because complete repeated outcome assessment data were unavailable.

#### 3.4.1 Interpretation of exploratory outcome analyses

Consistent with the exploratory design of the trial, the analyses presented below were undertaken to generate preliminary estimates of clinical and microbiological response. Accordingly, the observed between-group differences should be interpreted as exploratory and hypothesis-generating rather than confirmatory evidence of treatment efficacy.

#### 3.4.2 Exploratory primary outcome (objective parameter)

##### 3.4.2.1 Candida spore count

###### 3.4.2.1.1 Comparing the effect of treatment on candida spore count between group A and group B using the independent sample *t*-test

**Figure 2:**
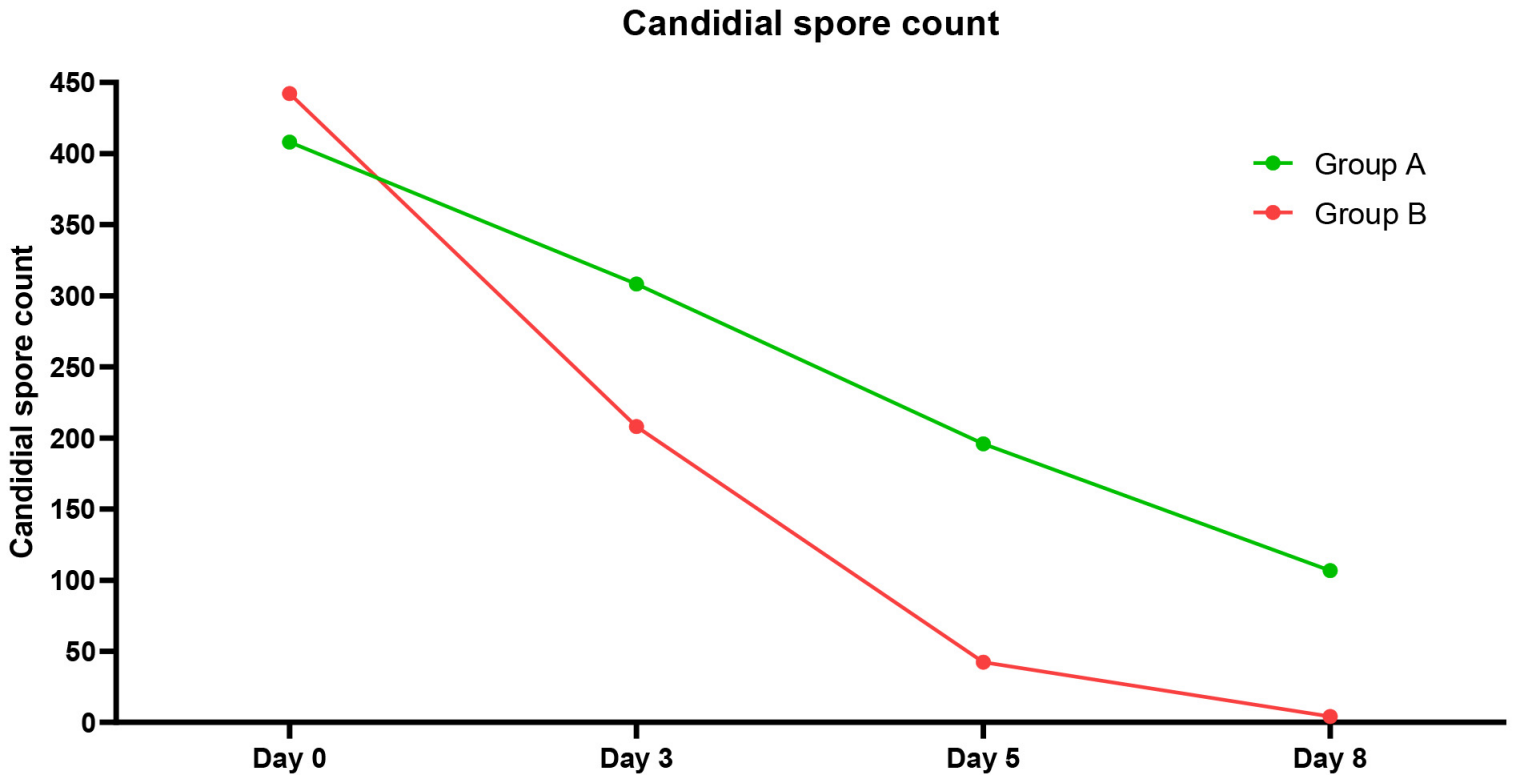
Candida spore count between group comparisons.

The between-group comparison of Candida spore counts at successive assessment time points is presented in Table 2 and Figure 2. Both groups demonstrated progressive reductions in fungal burden over the study period.

**Table 2:** Between-group comparison of Candida spore count (Independent sample *t*-test).

Time points	Candida spore count				mean difference	95% CI for mean diff	Effect size	Between group comparison (Unpaired *t* test)	
	Group A (*n* = 16)		Group B (*n* = 16)					*t*	*p*
	mean	SD	mean	SD					
Day 0	408.1	290.6	442.1	246.2	−34	−228.5–160.5	−0.126	−0.357	0.724
Day 3	308.2	217.6	208	113.1	100.2	−25–225.4	0.578	1.634	0.113
Day 5	195.9	134.9	42.4	41.3	153.5	81.5–225.5	1.539	4.352	0.001
Day 8	106.7	79.7	4.1	5.6	102.6	61.9–143.4	1.818	5.141	<.0001

At baseline (Day 0), the mean Candida spore counts were comparable between Group A (408.1 ± 290.6) and Group B (442.1 ± 246.2), with a mean difference of −34.0 (95% CI: −228.5 to 160.5). The effect size was negligible (Cohen's *d* = −0.126), indicating comparable baseline fungal burden between the groups (*t* = −0.357, *p* = 0.724).

By Day 3, both groups showed reductions in Candida spore count (Group A: 308.2 ± 217.6; Group B: 208.0 ± 113.1). The mean difference was 100.2 (95% CI: −25.0 to 225.4), with a moderate effect size (Cohen's *d* = 0.578). Although the confidence interval crossed zero, the findings suggested an early exploratory between-group difference favoring the intervention group (*t* = 1.634, *p* = 0.113).

A more pronounced between-group difference was observed on Day 5. Group B demonstrated a lower mean Candida spore count (42.4 ± 41.3) than Group A (195.9 ± 134.9), with a mean difference of 153.5 (95% CI: 81.5 to 225.5). The effect size was large (Cohen's *d* = 1.539), and the observed between-group difference was supported by the corresponding statistical comparison (*t* = 4.352, *p* < 0.001).

By Day 8, the mean Candida spore count further declined to 4.1 ± 5.6 in Group B compared with 106.7 ± 79.7 in Group A. The mean difference was 102.6 (95% CI: 61.9 to 143.4), with a very large effect size (Cohen's *d* = 1.818). The confidence interval remained entirely above zero, suggesting an exploratory between-group difference favoring the intervention group (*t* = 5.141, *p* < 0.0001).

###### 3.4.2.1.2 Effect of treatment on vaginal candidiasis in Candida spore count assessed using KOH wet mount and ImageJ software within group A and group B using repeated-measures ANOVA and Tukey's multiple comparison test

The Repeated-measures ANOVA was performed to evaluate changes in Candida spore count over time within and between the two treatment groups (Table 3). The analysis demonstrated a marked temporal reduction in Candida spore count throughout the study period. A significant main effect of time was observed [F_(1.29,38.8)_ = 70.6, *p* < 0.001], indicating progressive reductions in fungal burden across both treatment groups during follow-up.

**Table 3:** Repeated measures ANOVA for Candida spore reduction.

ANOVA table	SS	DF	MS	F (DFn, DFd)	*P* value
Time vs. Group	154,531	3	51,510	F_(3,90)_ = 4.23	*P* = 0.008
Time	2,581,774	3	860,591	F_(1.29,38.8)_ = 70.6	*P* < 0.001
Group	207,771	1	207,771	F_(1,30)_ = 2.62	*P* = 0.116

A significant time × group interaction was also observed (*F* = 4.23, *p* = 0.008), suggesting that the pattern and rate of reduction in Candida spore count differed between the two interventions over time. In contrast, the main effect of treatment group was not statistically significant (*F* = 2.62, *p* = 0.116), indicating comparable overall group means across the study period despite differences in the temporal pattern of response.

Overall, these exploratory analyses suggest that both interventions were associated with progressive reductions in Candida spore count over time, while the intervention group demonstrated an earlier reduction in fungal burden during follow-up.

Within Group A, Tukey's *post-hoc* multiple comparison analysis (Table 4) demonstrated progressive reductions in *Candida* spore count across all assessment time points.

**Table 4:** Tukey's multiple comparison test for group A.

Group A	Mean difference	SE	95% CI for mean diff.	Effect size (Cohen's d)	*p*
Day 0 vs. Day 3	99.9	23.1	50.6–149.2	1.08	0.003
Day 0 vs. Day 5	212	45.5	115.3–309.2	1.167	0.002
Day 0 vs. Day 8	301	54.3	185.6–417.2	1.387	0.001
Day 3 vs. Day 5	112	31.8	44.5–180.1	0.883	0.014
Day 3 vs. Day 8	202	35.3	126.3–276.7	1.427	0.001
Day 5 vs. Day 8	89.2	18.9	49–129.4	1.182	0.001

Compared with baseline (Day 0), statistically significant reductions were observed on Day 3 (mean difference = 99.9; 95% CI: 50.6 to 149.2; Cohen's *d* = 1.08; *p* = 0.003), Day 5 (mean difference = 212.0; 95% CI: 115.3 to 309.2; Cohen's *d* = 1.17; *p* = 0.002), and Day 8 (mean difference = 301.0; 95% CI: 185.6 to 417.2; Cohen's *d* = 1.39; *p* < 0.001), indicating progressive reductions in fungal burden over the treatment period.

Significant reductions were also observed between Day 3 and Day 5 (mean difference = 112.0; 95% CI: 44.5 to 180.1; Cohen's *d* = 0.88; *p* = 0.014), between Day 3 and Day 8 (mean difference = 202.0; 95% CI: 126.3 to 276.7; Cohen's *d* = 1.43; *p* < 0.001), and between Day 5 and Day 8 (mean difference = 89.2; 95% CI: 49.0 to 129.4; Cohen's *d* = 1.18; *p* = 0.001), suggesting continued microbiological improvement throughout the study period.

Overall, the exploratory analyses indicate that *Triphalā Kaṣāya Yoni Prakṣālana* was associated with progressive reductions in *Candida* spore count across the 7-day intervention period.

Within Group B, Tukey's post hoc multiple comparison analysis (Table 5) demonstrated progressive reductions in *Candida* spore count across all assessment time points.

**Table 5:** Tukey's multiple comparison test for group B.

Group B	Mean difference	SE	95% CI for mean diff.	Effect size (Cohen's d)	*p*
Day 0 vs. Day 3	234.1	43.4	141.7–326.6	1.349	<0.001
Day 0 vs. Day 5	399.8	56.5	279.4–520.1	1.77	<0.0001
Day 0 vs. Day 8	438.1	61.0	308.1–568	1.796	<0.0001
Day 3 vs. Day 5	165.6	21.8	119.2–212.1	1.9	<0.0001
Day 3 vs. Day 8	203.9	27.5	145.4–262.5	1.856	<0.0001
Day 5 vs. Day 8	38.3	9.3	18.4–58.2	1.025	0.005

Compared with baseline (Day 0), significant reductions in *Candida* spore count were observed on Day 3 (mean difference = 234.0; 95% CI: 141.7 to 326.6; Cohen's *d* = 1.35; *p* < 0.001), Day 5 (mean difference = 400.0; 95% CI: 279.4 to 520.1; Cohen's *d* = 1.77; *p* < 0.0001), and Day 8 (mean difference = 438.0; 95% CI: 308.1 to 568.0; Cohen's *d* = 1.80; *p* < 0.0001). These findings indicate substantial reductions in fungal burden during the intervention period.

Significant reductions were also observed between Day 3 and Day 5 (mean difference = 166.0; 95% CI: 119.2 to 212.1; Cohen's *d* = 1.90; *p* < 0.0001) and between Day 3 and Day 8 (mean difference = 204.0; 95% CI: 145.4 to 262.5; Cohen's *d* = 1.86; *p* < 0.0001), suggesting continued microbiological improvement following the initial reduction in fungal burden. The comparison between Day 5 and Day 8 also demonstrated a smaller but measurable reduction (mean difference = 38.3; 95% CI: 18.4 to 58.2; Cohen's *d* = 1.03; *p* = 0.005), indicating that reductions in *Candida* spore count continued through the end of the intervention.

Overall, the exploratory analyses suggest an earlier and greater reduction in *Candida* spore count in the intervention group during the study period. The clinical relevance of these observations should be interpreted cautiously and confirmed in future adequately powered confirmatory randomized controlled trials.

#### 3.4.3 Exploratory secondary outcome (subjective parameter)

##### 3.4.3.1 5-D pruritus score

**Figure 3:**
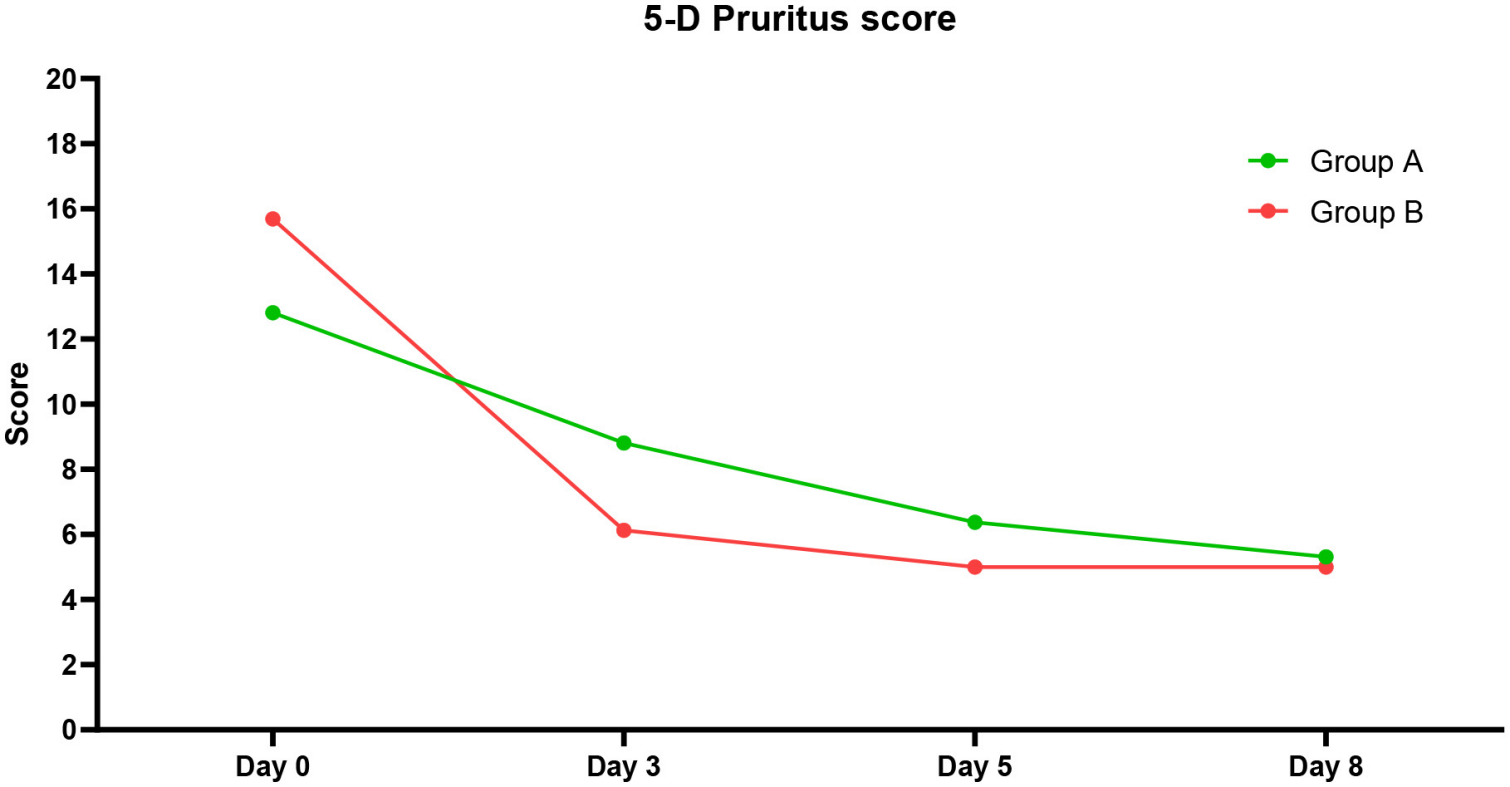
5-D pruritus score between-group comparison.

The between-group comparison of 5-D Pruritus scores at successive assessment time points is presented in Table 6 and Figure 3. Both treatment groups demonstrated progressive reductions in pruritus severity during the study period.

**Table 6:** Between-group comparison of 5-D pruritus scores (Independent sample *t*-test).

Time points	5-D pruritus				Mean difference	95% CI for mean diff	Effect size	Between group comparison (Unpaired *t* test)	
	Group A (*n* = 16)		Group B (*n* = 16)					*t*	*p*
	mean	SD	mean	SD					
Day 0	12.8	5.6	15.7	4.7	−2.9	−6.6–0.8	−0.56	1.585	0.124
Day 3	8.8	3.1	6.1	1.7	2.7	0.9–4.5	1.067	3.019	0.005
Day 5	6.4	1.5	5.0	0.0	1.4	0.6–2.1	1.337	3.78	0.001
Day 8	5.3	0.6	5.0	0.0	0.3	0–0.6	0.734	2.076	0.047

At baseline (Day 0), the mean 5-D Pruritus scores were comparable between Group A (12.8 ± 5.6) and Group B (15.7 ± 4.7), with a mean difference of −2.9 (95% CI: −6.6 to 0.8). Although the effect size was moderate (Cohen's *d* = −0.56), the confidence interval crossed zero, indicating comparable baseline pruritus severity between the groups (*t* = 1.585, *p* = 0.124).

By Day 3, both groups demonstrated reductions in pruritus scores, with Group B (6.1 ± 1.7) showing lower mean scores than Group A (8.8 ± 3.1). The mean difference was 2.7 (95% CI: 0.9 to 4.5), with a large effect size (Cohen's *d* = 1.067), suggesting an exploratory between-group difference favoring the intervention group (*t* = 3.019, *p* = 0.005).

On Day 5, Group A had a mean score of 6.4 ± 1.5 compared with 5.0 ± 0.0 in Group B. The mean difference was 1.4 (95% CI: 0.6 to 2.1), with a large effect size (Cohen's *d* = 1.337), suggesting a continued exploratory between-group difference (*t* = 3.780, *p* = 0.001).

By Day 8, mean pruritus scores further declined to 5.3 ± 0.6 in Group A and 5.0 ± 0.0 in Group B. The mean difference was 0.3 (95% CI: 0.0 to 0.6), with a large effect size (Cohen's *d* = 0.734). Although the magnitude of the between-group difference was smaller than that observed on Day 5, the exploratory comparison continued to favor the intervention group (*t* = 2.076, *p* = 0.047).

Overall, these exploratory analyses suggest earlier reduction in pruritus scores in the intervention group during the treatment period. These observations should be interpreted cautiously and require confirmation in adequately powered confirmatory randomized controlled trials.

###### 3.4.3.1.1 Effect of treatment on vaginal candidiasis in pruritus assessed through 5-D pruritus scale within group A & within group B using RM-ANOVA & Tukey's multiple comparison test

A repeated-measures ANOVA was performed to evaluate changes in 5-D Pruritus scores over time within and between the two treatment groups (Table 7). The analysis demonstrated a marked temporal reduction in pruritus scores throughout the study period. A significant main effect of time was observed [F_(1.35,40.5)_ = 90.0, *p* < 0.001], indicating progressive reductions in pruritus severity across both treatment groups during follow-up.

**Table 7:** Repeated measures ANOVA for pruritus score changes.

ANOVA table	SS	DF	MS	F (DFn, DFd)	*P* value
Time vs. Group	135	3	45.1	F_(3,90)_ = 7.22	*P* = 0.001
Time	1686	3	562	F_(1.35,40.5)_ = 90.0	*P* < 0.001
Group	10.1	1	10.1	F_(1,30)_ = 0.203	*P* = 0.656

A significant time × group interaction was also observed (*F* = 7.22, *p* = 0.001), suggesting that the pattern and rate of improvement differed between the two interventions over time. In contrast, the main effect of treatment group was not statistically significant (*F* = 0.203, *p* = 0.656), indicating comparable overall group means across the study period.

Overall, these exploratory analyses suggest that both interventions were associated with progressive reductions in pruritus scores over time, while the intervention group demonstrated earlier improvement during follow-up. These findings should be interpreted as exploratory and hypothesis-generating rather than as confirmatory evidence of treatment effectiveness.

Within Group A, Tukey's *post-hoc* multiple comparison analysis (Table 8) demonstrated progressive reductions in 5-D Pruritus scores across the study period.

**Table 8:** Tukey's multiple comparison test for group A.

Group A	Mean difference	SE	95% CI for mean diff.	Effect size (Cohen's d)	p
Day 0 vs. Day 3	4	0.842	2.21–5.79	1.188	0.001
Day 0 vs. Day 5	6.44	1.25	3.77–9.11	1.286	0.001
Day 0 vs. Day 8	7.5	1.32	4.68–10.32	1.417	0.000
Day 3 vs. Day 5	2.44	0.689	0.97–3.91	0.884	0.014
Day 3 vs. Day 8	3.5	0.736	1.93–5.07	1.189	0.001
Day 5 vs. Day 8	1.06	0.37	0.27–1.85	0.717	0.051

Compared with baseline (Day 0), significant reductions in pruritus scores were observed on Day 3 (mean difference = 4.00; 95% CI: 2.21 to 5.79; Cohen's *d* = 1.19; *p* = 0.001), Day 5 (mean difference = 6.44; 95% CI: 3.77 to 9.11; Cohen's *d* = 1.29; *p* = 0.001), and Day 8 (mean difference = 7.50; 95% CI: 4.68 to 10.32; Cohen's *d* = 1.42; *p* < 0.001), indicating progressive reductions in pruritus severity throughout the intervention period.

Significant reductions were also observed between Day 3 and Day 5 (mean difference = 2.44; 95% CI: 0.97 to 3.91; Cohen's *d* = 0.88; *p* = 0.014) and between Day 3 and Day 8 (mean difference = 3.50; 95% CI: 1.93 to 5.07; Cohen's *d* = 1.19; *p* = 0.001), suggesting continued improvement following the initial reduction in symptoms.

The comparison between Day 5 and Day 8 showed a smaller mean difference (1.06; 95% CI: 0.27 to 1.85; Cohen's *d* = 0.72), with a corresponding *p*-value of 0.051, suggesting that the magnitude of improvement had stabilized toward the end of the intervention period.

Overall, these exploratory analyses indicate progressive reductions in pruritus scores throughout the treatment period in participants receiving *Triphalā Kaṣāya Yoni Prakṣālana* alone.

Within Group B, Tukey's *post-hoc* multiple comparison analysis (Table 9) demonstrated progressive reductions in 5-D Pruritus scores across the study period.

**Table 9:** Tukey's multiple comparison test for group B.

Group B	Mean difference	SE	95% CI for mean diff.	Effect size (Cohen's d)	p
Day 0 vs. Day 3	9.56	1.06	7.3 - 11.8	2.263	0.001
Day 0 vs. Day 5	10.7	1.17	8.213.2	2.287	0.001
Day 0 vs. Day 8	10.7	1.17	8.213.2	2.287	0.001
Day 3 vs. Day 5	1.13	0.417	0.22	0.674	0.001
Day 3 vs. Day 8	1.13	0.417	0.22	0.674	0.001
Day 5 vs. Day 8	0			0	1.000

Compared with baseline (Day 0), significant reductions in pruritus scores were observed on Day 3 (mean difference = 9.56; 95% CI: 7.3 to 11.8; Cohen's *d* = 2.26; *p* = 0.001), Day 5 (mean difference = 10.7; 95% CI: 8.2 to 13.2; Cohen's *d* = 2.29; *p* = 0.001), and Day 8 (mean difference = 10.7; 95% CI: 8.2 to 13.2; Cohen's *d* = 2.29; *p* = 0.001), indicating substantial reductions in pruritus severity during the intervention period.

Significant reductions were also observed between Day 3 and Day 5 (mean difference = 1.13; Cohen's *d* = 0.67; *p* = 0.017) and between Day 3 and Day 8 (*p* = 0.001), suggesting continued improvement following the initial reduction in symptoms. No appreciable difference was observed between Day 5 and Day 8 (*p* = 1.000), indicating stabilization of pruritus scores during the latter part of the follow-up period.

Overall, these exploratory analyses suggest earlier reductions in pruritus scores in participants receiving the combination of *Triphalā Kaṣāya* and *Gomūtra Arka* during the study period. These observations should be interpreted as exploratory and hypothesis-generating and require confirmation in adequately powered confirmatory randomized controlled trials.

##### 3.4.3.2 Per vaginal discharge

###### 3.4.3.2.1 Comparing the effect of treatment on vaginal candidiasis in per vaginal discharge assessed through discharge scale between group A & group B using the Mann-Whitney *U* test

**Figure 4:**
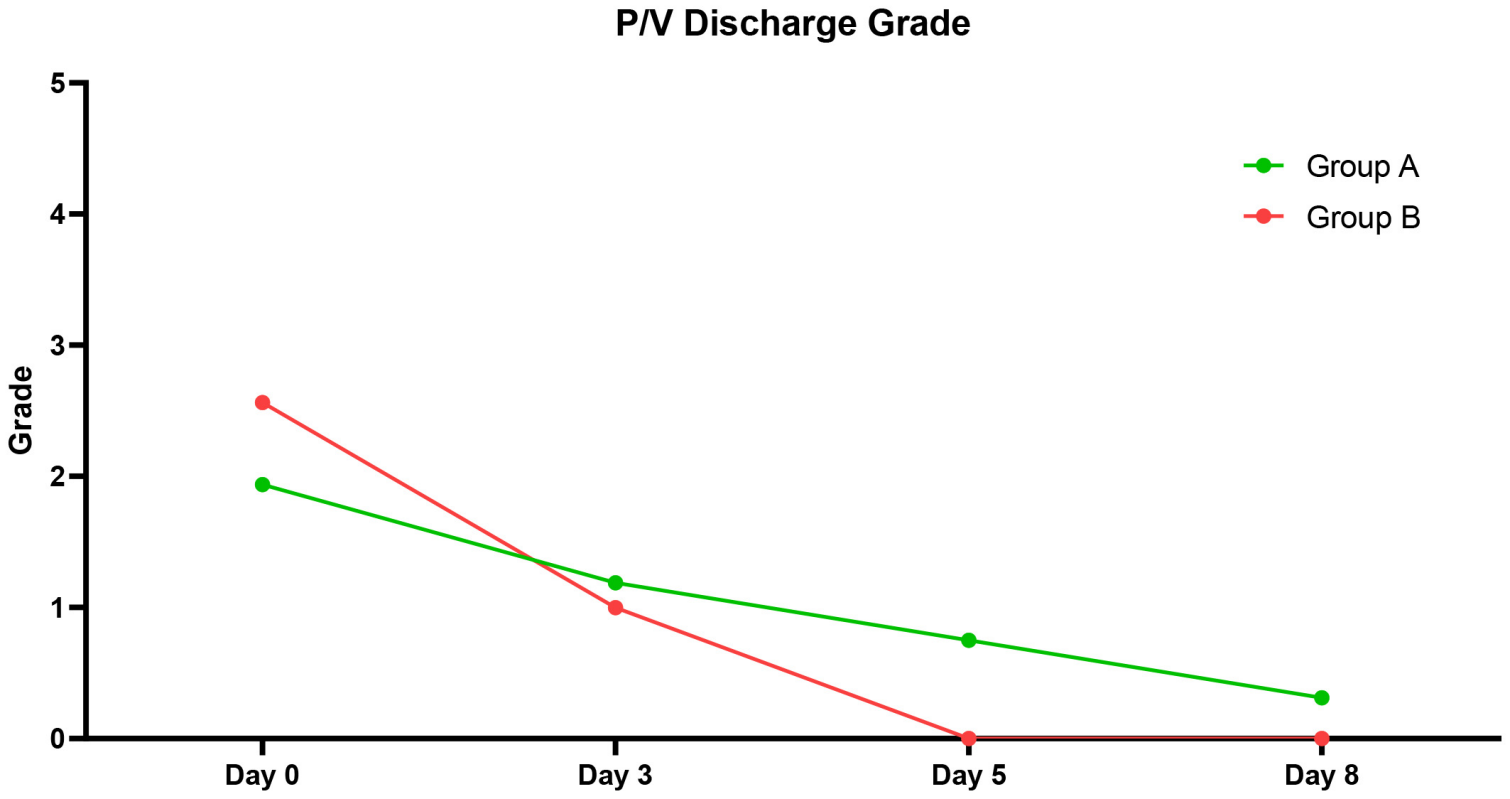
Discharge scale between-group comparison.

The between-group comparison of per vaginal discharge grades at successive assessment time points is presented in Table 10 and Figure 4. Both treatment groups demonstrated progressive reductions in discharge severity over the study period.

**Table 10:** Between-group comparison of P/V discharge grade (Mann–Whitney *U*-test).

Time points	P/V discharge grade				Median difference	95% CI for median diff	Effect size	Between-group comparison (Mann-Whitney *U*-test)	
	Group A (*n* = 16)		Group B (*n* = 16)						
	Median	IQR	Median	IQR				*U*	*p*
Day 0	2	1.75–2	3	2–3	−1	−1–0	0.484	66	0.011
Day 3	1	1–2	1	1–1	0	0–1	−0.152	108.5	0.415
Day 5	1	0.75–1	0	0–0	1	1–1	−0.75	32	<0.001
Day 8	0	0–1	0	0–0	0	0–0	−0.313	88	0.018

At baseline (Day 0), participants in Group B had a higher median discharge grade (Median = 3, IQR = 2–3) than those in Group A (Median = 2, IQR = 1.75–2). The median difference was −1 (95% CI: −1 to 0), with a moderate effect size (0.484), indicating a baseline difference between the groups (*U* = 66, *p* = 0.011).

By Day 3, both groups demonstrated marked reductions in discharge severity, with median scores converging to 1 (Group A: IQR = 1–2; Group B: IQR = 1–1). The median difference was 0 (95% CI: 0 to 1), with a negligible effect size (−0.152), suggesting no appreciable between-group difference at this assessment (*U* = 108.5, *p* = 0.415).

On Day 5, Group B demonstrated a median discharge grade of 0 (IQR = 0–0), whereas Group A had a median grade of 1 (IQR = 0.75–1). The median difference was 1 (95% CI: 1 to 1), with a large effect size (−0.75), suggesting an exploratory between-group difference favoring the intervention group (*U* = 32, *p* < 0.001).

By Day 8, both groups demonstrated further improvement, although Group B maintained lower discharge grades (Median = 0, IQR = 0–0) than Group A (Median = 0, IQR = 0–1). The median difference was 0 (95% CI: 0 to 0), with a moderate effect size (−0.313), suggesting a continued exploratory between-group difference (*U* = 88, *p* = 0.018).

Overall, these exploratory analyses suggest earlier reduction in vaginal discharge grades in the intervention group during the treatment period. These observations should be interpreted cautiously and confirmed in future adequately powered confirmatory randomized controlled trials.

###### 3.4.3.2.2 Effect of treatment on vaginal candidiasis in per vaginal discharge assessed through discharge scale within group A & within group B using the Friedman test with Durbin-Conover *post-hoc* comparisons

The longitudinal analysis of per vaginal discharge grades within Group A was performed using the Friedman test to evaluate changes across repeated assessment time points (Table 11). A significant overall reduction in discharge grades was observed during the study period (χ^2^ = 32.902, df = 3, *p* < 0.001), indicating progressive improvement over time.

Table 11Overall longitudinal change in P/V discharge grade within group A (Friedman Test with Durbin–Conover *post-hoc* comparisons).

**Table:** 

Overall change (Friedman Test)	χ^2^	df	*p*
**Group A**	32.902	3	<0.001

**Table:** 

Pairwise comparisons - group A	Median difference	SE of difference	95% CI for median difference	Effect size	Durbin-Conover	
					Statistic	*p*
Day 0 vs. Day 3	1	0.112	–	0.147	4.602	<0.001
Day 0 vs. Day 5	1.5	0.188	1–2	0.338	7.039	<0.001
Day 0 vs. Day 8	2	0.239	1.5–2.5	0.544	9.475	<0.001
Day 3 vs. Day 5	1	0.157	1–1	−0.412	2.436	0.019
Day 3 vs. Day 8	1	0.221	1–1.5	0.081	4.873	<0.001
Day 5 vs. Day 8	1	0.128	–	−0.588	2.436	0.019

Durbin–Conover post hoc comparisons demonstrated significant reductions between baseline (Day 0) and Day 3 (median difference = 1; effect size = 0.147; Statistic = 4.602; *p* < 0.001), Day 5 (median difference = 1.5; 95% CI: 1.0 to 2.0; effect size = 0.338; Statistic = 7.039; *p* < 0.001), and Day 8 (median difference = 2.0; 95% CI: 1.5 to 2.5; effect size = 0.544; Statistic = 9.475; *p* < 0.001), suggesting progressive reductions in discharge grades throughout the intervention period.

Significant reductions were also observed between Day 3 and Day 5 (median difference = 1; 95% CI: 1.0 to 1.0; Statistic = 2.436; *p* = 0.019) and between Day 3 and Day 8 (median difference = 1; 95% CI: 1.0 to 1.5; Statistic = 4.873; *p* < 0.001), indicating continued improvement following the initial reduction in symptoms. The comparison between Day 5 and Day 8 remained statistically significant (*p* = 0.019), suggesting that reductions in discharge grades continued until the end of the intervention period.

Overall, these exploratory analyses indicate progressive reductions in per vaginal discharge grades throughout the study period in participants receiving *Triphalā Kaṣāya Yoni Prakṣālana* alone.

The longitudinal analysis of per vaginal discharge grades within Group B was performed using the Friedman test to evaluate changes across repeated assessment time points (Table 12). A significant overall reduction in discharge grades was observed during the study period (χ^2^ = 44.91, df = 3, *p* < 0.0001), indicating marked improvement over time.

Table 12Overall longitudinal change in P/V discharge grade within group B (Friedman Test with Durbin–Conover *post-hoc* Comparisons).

**Table:** 

Overall change (Friedman test)	χ^2^	df	*p*
**Group B**	44.91	3	<0.0001

**Table:** 

Pairwise comparisons - group B	Median difference	SE of difference	95% CI for median difference	Effect size	Durbin-Conover	
					Statistic	*p*
Day 0 vs. Day 3	1.5	0.2	1.5–2	0.765	10.279	<0.001
Day 0 vs. Day 5	2.5	0.1	2.5–3	1	21.7	<0.001
Day 0 vs. Day 8	2.5	0.1	2.5–3	1	21.7	<0.001
Day 3 vs. Day 5	1	0.2	1–1.5	0.338	11.421	<0.001
Day 3 vs. Day 8	1	0.2	1–1.5	0.338	11.421	<0.001
Day 5 vs. Day 8		0	–	−1	0	1

Durbin–Conover post hoc comparisons demonstrated significant reductions between baseline (Day 0) and Day 3 (median difference = 1.5; 95% CI: 1.5 to 2.0; effect size = 0.765; Statistic = 10.279; *p* < 0.001), Day 5 (median difference = 2.5; 95% CI: 2.5 to 3.0; effect size = 1.000; Statistic = 21.700; *p* < 0.001), and Day 8 (median difference = 2.5; 95% CI: 2.5 to 3.0; effect size = 1.000; Statistic = 21.700; *p* < 0.001), indicating progressive reductions in discharge grades during follow-up.

Significant reductions were also observed between Day 3 and Day 5 (median difference = 1; 95% CI: 1.0 to 1.5; effect size = 0.338; Statistic = 11.421; *p* < 0.001) and between Day 3 and Day 8 (*p* < 0.001), suggesting continued improvement following the initial reduction in discharge grades. No appreciable difference was observed between Day 5 and Day 8 (Statistic = 0; *p* = 1.000), indicating stabilization of discharge grades during the latter part of the follow-up period.

Overall, these exploratory analyses suggest earlier reductions in per vaginal discharge grades in participants receiving the combination of *Triphalā Kaṣāya* and *Gomūtra Arka* during the intervention period. These observations should be interpreted as exploratory and hypothesis-generating and require confirmation in adequately powered confirmatory randomized controlled trials.

### 3.5 Harms

Throughout the intervention period, participants were prospectively monitored for adverse events using the Naranjo Adverse Drug Reaction Probability Scale. No intervention-related serious adverse events or clinically significant safety concerns were identified in either treatment group. Both interventions were well tolerated. Four participants discontinued treatment because of personal reasons.

### 3.6 Ancillary analyses

No subgroup analyses were prespecified or performed due to the exploratory nature and limited sample size of the study. No sensitivity analyses were conducted. All reported analyses were prespecified in the study protocol and statistical analysis plan.

For this exploratory randomized trial, the observed effect estimates and confidence intervals are intended primarily to inform the design, feasibility assessment, and sample size estimation of future definitive randomized controlled trials.

The present study was designed as an exploratory randomized clinical trial to generate preliminary estimates of clinical and microbiological outcomes and to inform the design of future confirmatory randomized controlled trials. Accordingly, the analyses presented above should be interpreted as exploratory and hypothesis-generating rather than confirmatory. Although descriptive statistical comparisons, effect size estimates, confidence intervals, and *p*-values are presented to facilitate interpretation of the observed data patterns, the study was not designed or powered to establish definitive therapeutic efficacy or effectiveness. Therefore, the observed between-group differences should be interpreted with appropriate caution and considered as preliminary evidence requiring confirmation in larger, adequately powered multicentre randomized controlled trials.

## 4 Discussion

The present study was designed as an exploratory randomized clinical trial to assess the feasibility, safety, and preliminary clinical and microbiological outcomes of adding *Gomūtra Arka* to *Triphalā Kaṣāya Yoni Prakṣālana* (vaginal douche) in women with vaginal candidiasis (*Ślaiṣmikī Yonivyāpat*). Rather than providing confirmatory evidence of therapeutic efficacy, the trial was intended to generate preliminary, hypothesis-generating observations that may inform the design, outcome selection, and sample size estimation of future adequately powered confirmatory randomized controlled trials.

Vaginal candidiasis remains one of the most common fungal infections affecting women of reproductive age and is frequently associated with considerable discomfort, impaired quality of life, recurrent episodes, and emerging concerns regarding antifungal resistance. These challenges highlight the need to explore safe, evidence-informed complementary therapeutic approaches. Accordingly, the present study was designed to evaluate whether the addition of Gomutra Arka could optimize an existing Ayurvedic local therapeutic intervention, namely Triphalā Kaṣāya Yoni Prakṣālana, rather than replace conventional antifungal therapy. Therefore, the findings should be interpreted within an integrative therapeutic framework and not as evidence of comparative efficacy against standard antifungal agents.

Although participant and treating investigator blinding was not feasible because of the characteristic properties of Gomutra Arka, measures were undertaken to minimize assessment bias through independent evaluation of subjective clinical outcomes and objective ImageJ-assisted quantification of Candida spores. In addition, baseline demographic and clinical characteristics were generally comparable between the intervention and control groups, supporting the internal validity of the exploratory comparisons while recognizing that the findings should be interpreted cautiously in view of the exploratory design.

### 4.1 Discussion of clinical and microbiological outcomes

#### 4.1.1 Effect on Candida spore count

Baseline *Candida* spore counts were comparable between the two treatment groups, indicating similar microbiological fungal burden before initiation of the interventions. Both treatment groups demonstrated progressive reductions in *Candida* spore count during the study period, suggesting that *Triphalā Kaṣāya Yoni Prakṣālana* may itself contribute to microbiological improvement in women with vaginal candidiasis.

Exploratory between-group analyses suggested earlier and greater reductions in *Candida* spore count among participants receiving the combination of *Triphalā Kaṣāya* and *Gomūtra Arka* compared with *Triphalā Kaṣāya* alone. This pattern was supported by the repeated-measures ANOVA, which demonstrated a significant effect of time together with a significant time × group interaction, indicating that the temporal pattern of fungal reduction differed between the two interventions. Although the present exploratory study was not designed to establish comparative therapeutic efficacy, these findings suggest that the addition of *Gomūtra Arka* may be associated with earlier microbiological improvement during the short-term intervention period.

The biological mechanisms underlying these observations were not investigated in the present clinical trial. However, previous experimental studies have demonstrated that *Triphalā* possesses antifungal, antioxidant, and anti-inflammatory properties, while Gomutra Arka has shown antifungal activity against *Candida* species together with potential bioenhancing effects. These experimental observations provide biological plausibility for the exploratory findings of the present study but should not be interpreted as confirmation of the underlying mechanisms. Further mechanistic, microbiological, and translational studies are required to determine whether these pharmacological properties contribute to the earlier microbiological improvement observed with the combination therapy.

#### 4.1.2 Effect on pruritus

Pruritus is one of the most distressing symptoms of vaginal candidiasis and contributes substantially to impaired patient comfort and quality of life. Baseline 5-D Pruritus Scale scores were comparable between the two treatment groups, indicating similar symptom severity before treatment initiation.

Both treatment groups demonstrated progressive reductions in pruritus scores throughout the intervention period. Exploratory between-group analyses suggested earlier improvement among participants receiving *Triphalā Kaṣāya* combined with *Gomūtra Arka* compared with those receiving *Triphalā Kaṣāya* alone. Repeated-measures ANOVA demonstrated a significant effect of time together with a significant time × group interaction, suggesting that the temporal pattern of symptom improvement differed between the two interventions. Although the present exploratory study was not designed to establish comparative therapeutic effectiveness, these findings suggest that the addition of *Gomūtra Arka* may be associated with earlier symptomatic improvement during the short-term intervention period.

The earlier reduction in pruritus observed in the intervention group may be related to more rapid reduction in local fungal burden together with the anti-inflammatory properties traditionally attributed to the intervention components in Ayurvedic literature. However, these potential mechanisms were not investigated in the present study and therefore remain hypothetical. Further mechanistic investigations and adequately powered confirmatory clinical trials are required to determine whether these observations can be reproduced and to better understand the biological basis of the observed clinical response.

#### 4.1.3 Effect on vaginal discharge

Reduction in abnormal vaginal discharge is an important clinical outcome in the management of vaginal candidiasis, as it reflects improvement in both patient symptoms and local disease activity. Unlike the other baseline outcome measures, participants in the intervention group presented with slightly higher vaginal discharge grades at baseline. This imbalance is likely attributable to the relatively small sample size and the use of simple randomization, both of which may result in chance differences between treatment groups in exploratory clinical trials. Accordingly, findings related to vaginal discharge should be interpreted with appropriate caution.

Despite this baseline imbalance, both treatment groups demonstrated progressive reductions in vaginal discharge during the intervention period. Exploratory between-group analyses suggested earlier improvement among participants receiving *Triphalā Kaṣāya* combined with *Gomūtra Arka* compared with those receiving *Triphalā Kaṣāya* alone. Within-group longitudinal analyses similarly demonstrated progressive reductions in discharge grades over time in both groups, with the intervention group showing an earlier reduction during follow-up. However, because of the baseline difference in discharge severity, these observations should be regarded as preliminary and hypothesis-generating rather than confirmatory evidence of comparative therapeutic effectiveness.

The earlier reduction in vaginal discharge observed in the intervention group may be biologically plausible in view of the reported antimicrobial, anti-inflammatory, and potential bioenhancing properties of *Triphalā* and *Gomūtra Arka* described in previous experimental studies. Nevertheless, these mechanisms were not evaluated in the present trial and therefore remain speculative. Future adequately powered multicentre randomized controlled trials incorporating longer follow-up and objective microbiological assessment are required to determine whether these preliminary observations can be confirmed.

#### 4.1.4 Overall therapeutic response

Both treatment groups were associated with progressive improvements in microbiological and clinical outcome measures over the study period. Exploratory between-group analyses suggested earlier reductions in *Candida* spore count, pruritus, and vaginal discharge among participants receiving *Triphalā Kaṣāya* combined with *Gomūtra Arka* compared with *Triphalā Kaṣāya* alone. Corresponding statistical comparisons indicated that these between-group differences became apparent from Day 5 onward and were maintained through the Day 8 assessment.

The present study was designed to evaluate immediate short-term clinical response and early microbiological changes following a standardized 7-day intervention schedule. Consequently, the findings primarily reflect short-term treatment response and should not be interpreted as evidence of sustained remission, long-term disease control, prevention of recurrence, or definitive comparative therapeutic effectiveness. In addition to the observed preliminary clinical and microbiological improvements, no intervention-related serious adverse events or clinically significant safety concerns were identified during the study period, supporting the short-term safety of the intervention under the conditions evaluated in this exploratory trial.

Taken together, these exploratory findings suggest that the addition of *Gomūtra Arka* to *Triphalā Kaṣāya Yoni Prakṣāla*na may provide additional short-term clinical and microbiological benefit. However, given the exploratory design, relatively small sample size, single-center setting, and limited follow-up duration, these observations should be regarded as hypothesis-generating and require confirmation in adequately powered multicentre randomized controlled trials with longer follow-up before definitive conclusions regarding comparative therapeutic effectiveness can be drawn.

### 4.2 Probable mode of action *Triphalā Kaṣāya* and *Gomūtra Arka*

*Yoni Prakṣālana* (vaginal douche) is described in Ayurveda as a local cleansing (*Sthānika Sodhana*) procedure intended to act directly at the site of pathology. The combination of *Triphalā Kaṣāya* and *Gomūtra Arka* has traditionally been recommended in the management of *Srāva-pradhāna Ślaiṣmikī Yonivyāpat*, suggesting a complementary therapeutic approach for conditions characterized predominantly by abnormal vaginal discharge.

*Triphalā* is a classical polyherbal formulation comprising *Harītakī* (*Terminalia chebula*), *Vibhītakī* (*Terminalia bellirica*), and Ā*malakī* (*Emblica officinalis*). Experimental studies have demonstrated that *Triphalā* contains bioactive phytochemicals, including tannins, gallic acid, ellagic acid, flavonoids, and other phenolic compounds, which exhibit antifungal, antioxidant, anti-inflammatory, and immunomodulatory activities against *Candida albicans* and other microorganisms. These pharmacological properties provide a biologically plausible explanation for the progressive microbiological and clinical improvements observed in both treatment groups (25).

Compared with *Triphalā Kaṣāya* alone, the combination therapy produced earlier reductions in *Candida* spore count, pruritus, and vaginal discharge. This enhanced response may be associated with the pharmacological properties traditionally attributed to *Gomūtra Arka*, including *Krimighna, Kandughna*, Ś*ophahara*, and *Kapha-Vāta Hara* actions.

*Gomūtra Arka* has also been investigated experimentally for its antimicrobial properties. Previous *in vitro* studies have demonstrated antifungal activity against *Candida* species, while chromatographic analyses have identified several phenolic compounds that may contribute to these effects (17). In addition, experimental *in silico* studies have suggested that certain bioactive constituents may interact with microbial proteins and influence membrane integrity, although these findings remain preliminary and require further biological validation (26).

Another characteristic of *Gomūtra Arka* that has received scientific attention is its potential bioenhancing activity. Experimental evidence suggests that cow urine distillate may enhance the cellular uptake of selected antimicrobial and other therapeutic agents, thereby improving their biological availability (27). Although this mechanism has been demonstrated primarily under experimental conditions, it provides one possible explanation for the earlier microbiological and clinical improvements observed in participants receiving the combination therapy in the present study.

However, it is important to emphasize that the present exploratory randomized clinical trial was designed to evaluate preliminary clinical and microbiological outcomes and did not investigate pharmacological mechanisms. Therefore, the proposed antifungal, anti-inflammatory, immunomodulatory, and bioenhancing actions should be regarded only as biologically plausible explanations derived from previous experimental evidence and classical Ayurvedic literature. Confirmation of these mechanisms will require dedicated mechanistic studies incorporating microbiological, molecular, metabolomic, and pharmacodynamic investigations.

## 5 Conclusion

This exploratory randomized clinical trial provides preliminary, hypothesis-generating evidence suggesting that the addition of *Gomūtra Arka* to *Triphalā Kaṣāya Yoni Prakṣālana* may be associated with earlier improvement in clinical symptoms, including pruritus and vaginal discharge, together with faster microbiological clearance of *Candida* spores compared with *Triphalā Kaṣāya Yoni Prakṣālana* alone in women with vaginal candidiasis (*Ślaiṣmikī Yonivyāpat*). Both interventions were feasible, well-tolerated, and demonstrated favorable exploratory clinical and microbiological trends over the study period.

However, the findings should be interpreted with appropriate caution in view of the exploratory design, single-center setting, relatively small sample size, and short follow-up duration. Consequently, the observed between-group differences should be regarded as preliminary estimates intended to inform future research rather than as definitive evidence of therapeutic efficacy or effectiveness. Larger adequately powered multicentre randomized controlled trials with longer follow-up are warranted to confirm these findings, evaluate recurrence patterns, establish long-term safety, and determine the clinical effectiveness of the combined intervention.

### 5.1 Limitations

The present study has several limitations that should be considered when interpreting the findings. As an exploratory randomized clinical trial, the study was designed to generate preliminary clinical and microbiological estimates rather than provide confirmatory evidence of therapeutic efficacy or effectiveness. Consequently, the observed between-group differences should be interpreted as hypothesis-generating and intended to inform the design of future adequately powered confirmatory randomized controlled trials rather than as definitive evidence of treatment benefit.

The study was conducted at a single center with a relatively small sample size, which may limit the precision of the estimated treatment effects and the generalizability of the findings. Although exploratory analyses identified between-group differences over time, the study was not designed or statistically powered to establish definitive treatment effects or clinical superiority.

The absence of a standard antifungal comparator limits direct comparison with conventional treatment strategies and therefore restricts broader clinical applicability. In addition, microbiological diagnosis relied on KOH wet mount microscopy without fungal culture, species-level identification, or molecular confirmation. Although KOH microscopy was selected because of its feasibility, rapid point-of-care applicability, and suitability for repeated short-interval assessments within the exploratory study design, more advanced microbiological methods would have improved diagnostic precision and enabled evaluation of species-specific therapeutic responses.

The open-label design may have introduced performance and assessment bias, particularly for subjective outcomes such as pruritus and vaginal discharge. To minimize this risk, subjective clinical outcomes were assessed by an independent assessor who was not involved in intervention delivery, while microbiological outcomes were independently evaluated by a microbiologist who had no role in participant recruitment, randomization, intervention administration, or clinical assessment. Furthermore, *Candida* spore quantification was performed using ImageJ-assisted image analysis to enhance measurement objectivity and reduce observer-dependent variability. Nevertheless, the absence of complete blinding remains an important methodological limitation.

The primary analyses followed a per-protocol approach because participants who discontinued the intervention did not complete the scheduled repeated outcome assessments required for longitudinal analysis. Although this approach was considered appropriate for estimating preliminary intervention effects in an exploratory trial, it may increase susceptibility to attrition bias compared with an intention-to-treat analysis.

Finally, the short follow-up period prevented evaluation of recurrence rates, sustained clinical improvement, long-term microbiological remission, delayed adverse events, and long-term safety, all of which are clinically important considerations in recurrent vulvovaginal candidiasis. In addition, participants were recruited from a single geographical and institutional setting, which may limit the external validity of the findings across populations with different demographic characteristics, healthcare systems, and environmental or microbiological profiles.

### 5.2 Future perspectives

Future multicentric, adequately powered randomized controlled trials with larger sample sizes and extended follow-up durations are required to validate the present findings and assess long-term outcomes, including recurrence rates and sustained microbiological remission and safety.

Future comparative studies incorporating standard antifungal agents as active comparators would further help to establish the relative therapeutic effectiveness of these interventions within broader clinical practice.

Further microbiome-based and translational studies evaluating changes in vaginal microbial composition, Lactobacillus dominance, local inflammatory markers, and host immune markers, and the antifungal and potential bioenhancing mechanisms of *Gomūtra Arka* are required to provide deeper mechanistic insights into the observed clinical benefits.

## Data Availability

The de-identified individual participant data underlying the findings of this study will be made available by the corresponding author upon reasonable request. Shared materials may include the anonymized participant-level dataset, data dictionary, and statistical analysis code, where applicable. Access to the data will be granted for legitimate academic and scientific research purposes, subject to institutional policies, ethical approval requirements, and applicable data protection and confidentiality regulations.
