# Supplementary material for: Exploratory randomized open-label clinical trial of Triphalā Kaṣāya vaginal douche (Yoni prakṣālana) with and without Gomūtra Arka in vaginal candidiasis
Source: Front Med (Lausanne). 2026 Aug 13;13:1910053. doi: 10.3389/fmed.2026.1910053 (PMC13527936; doi:10.3389/fmed.2026.1910053)
Supplement: Supplementary file 1 [file Data_Sheet_1.pdf]

ACARA
AMRITA

Amrita Centre for Advanced Research in Ayurveda

Amrita School of Ayurveda Campus
Clappana.P.O, Vallikavu, Kollam, Kerala-690525, India
Ph: +91-476-2899688 |mail: acaralab@ay.amrita.edu

ANTI-MICROBIAL ACTIVITY TESTING REPORT

- Customer Details : Dr.C.Roshini, Dept of Prasootitantra, ASA
- Test Requisition No. & Date : ACARA065/23-24, 10/03/24
- Number of samples : 3
- Date of Sample Received : 10/3/24
- Sample Analysis Date : 10/3/24 to 12/3/24
- Sample Name :different ratios of Triphala Kashayam(TK), Gomutra Arkam Prepared (GAP) and Gomutra arkam market sample (GAM)
- Sample ID : Nil
- Name of Manufacturer : Not mentioned
- Manufacturing License no : Not mentioned
- Batch No : Not mentioned
- Date of Manufacturing : Not mentioned
- Date of Expiry : Not mentioned
- Quantity of Sample Received : 10ml each
- Samples condition/nature : Good

Aim

To understand the antimicrobial activity of the samples in fungi *Candida albicans.*

Study Details

Materials Required

Saboraud Dextrose Agar (Merck, Germany), Micropipettes, Autoclaved Pipette tips, Petri plates, Conical Flask, L-rod, Spirit, Bunsen burner, Autoclaved MilliQ water, Discard beaker, Autoclaved microcentrifuge tubes.

Protocol

- Prepare SDA plates, Inoculate plates with the test microorganism (50 µl Candida albicans inoculum from previously prepared broth) by spread plate method.
- Prepare 10ml each of the test samples by mixing different ratios Triphala Kashayam (TK), Gomutra Arkam Prepared (GAP) and Gomutra arkam market sample (GAM). The ratios are 900:100, 850:150, 800:200 and 750:250 of TK and GA respectively.
- Make agar wells of approximately 10mm in diameter and fill them by adding the samples under test to the concerned wells. Each well should be made in such a way that the antibacterial zone of the drug added is easily visible in the agar surface and distributed evenly so that they are no closer than 24 mm from each other, center to center..
- Incubate the SDA plates for 48 hours inside an incubator. After incubation, examine all plates under test.
- The resulting zones of inhibition will be uniformly circular with a confluent lawn of growth. Measure the diameters of the zones of complete inhibition including the diameter of control zone.

Results

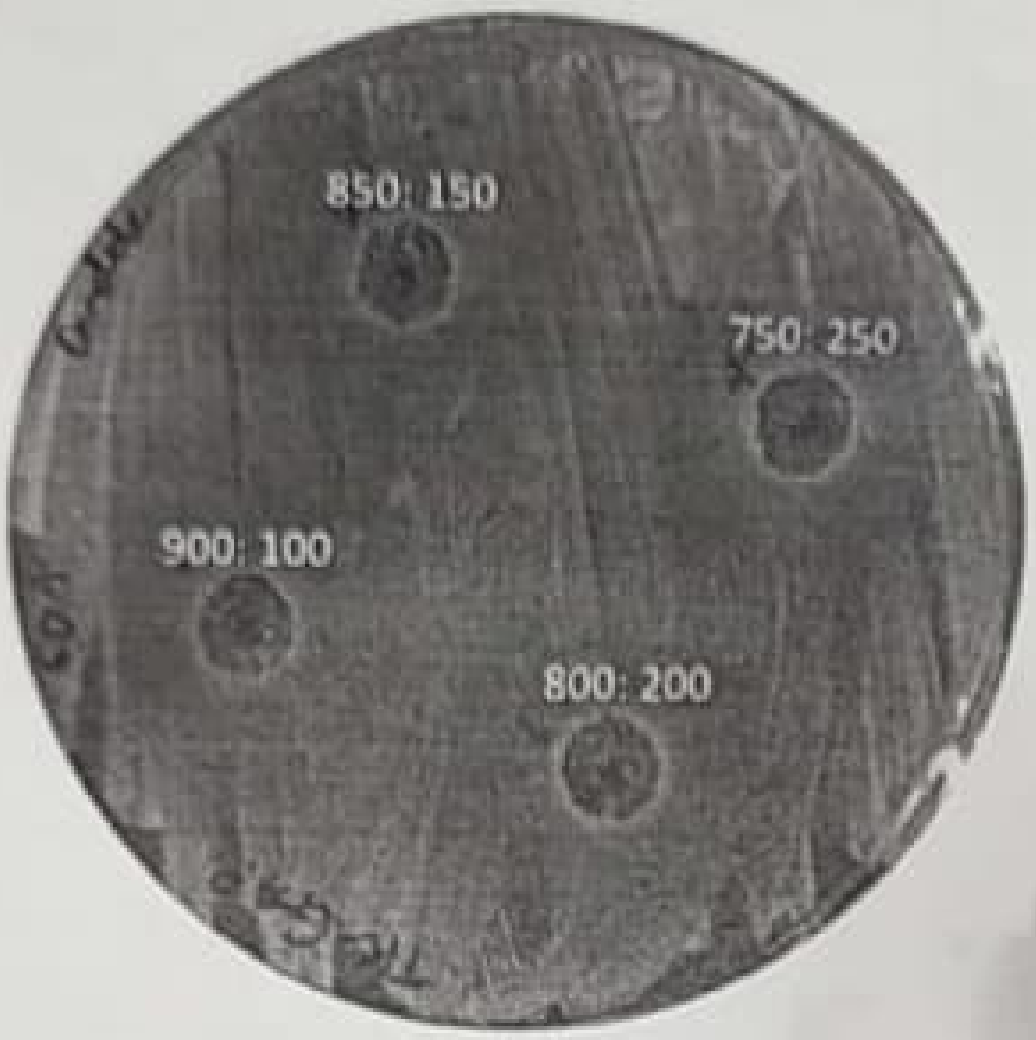

(a)

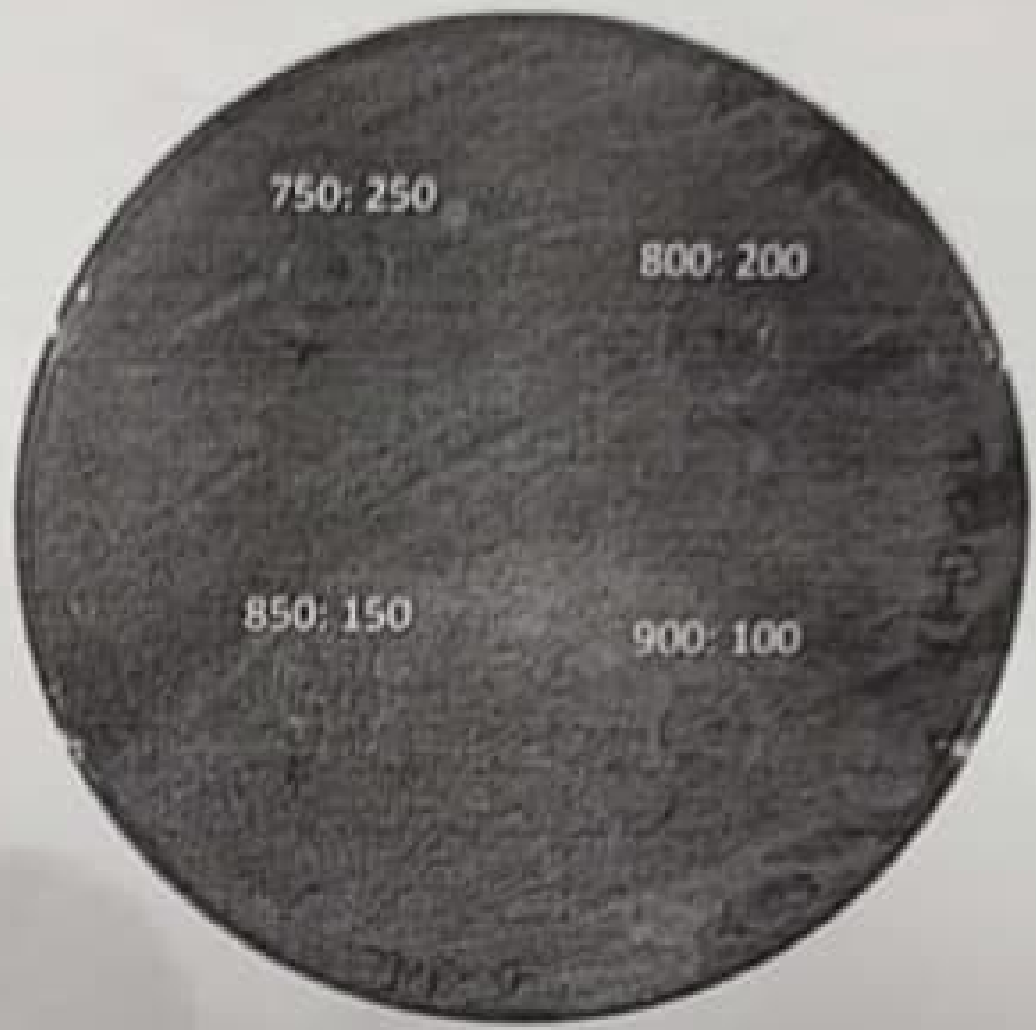

(b)

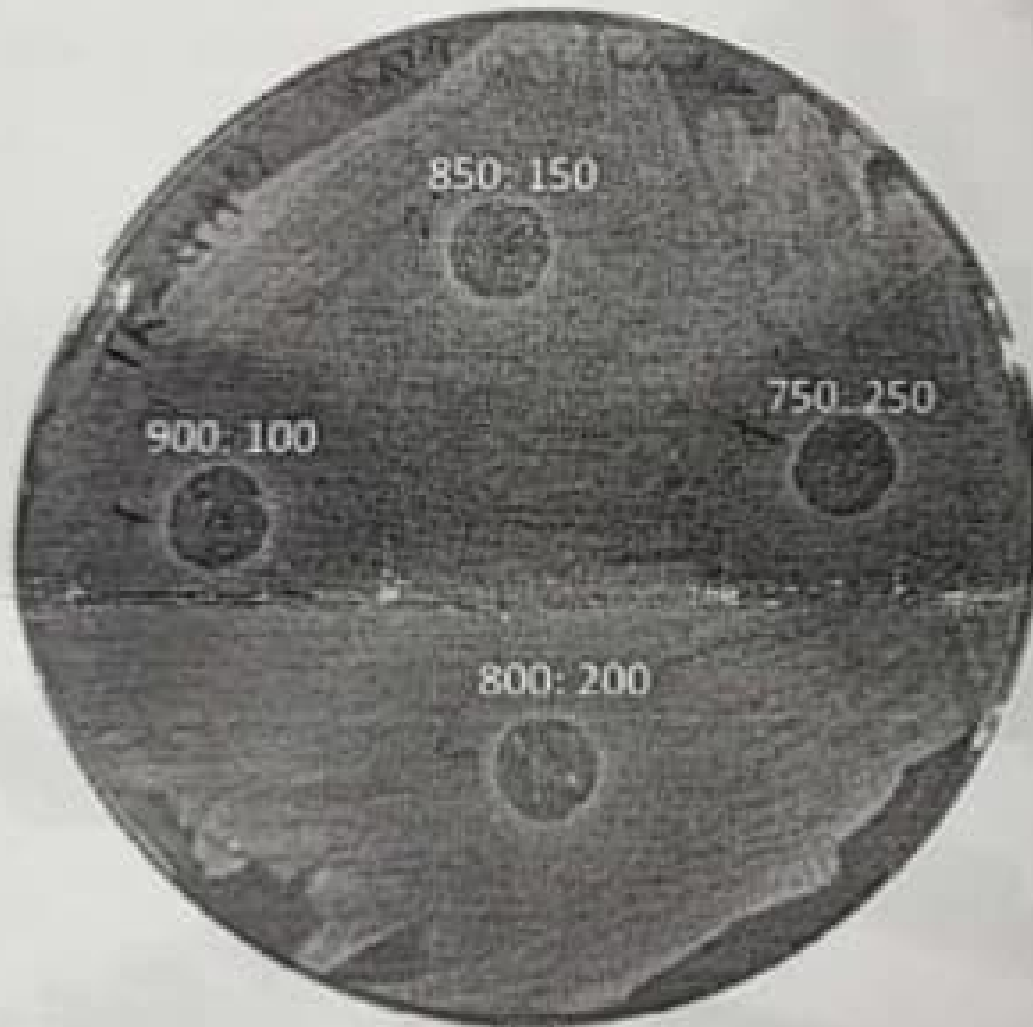

(c)

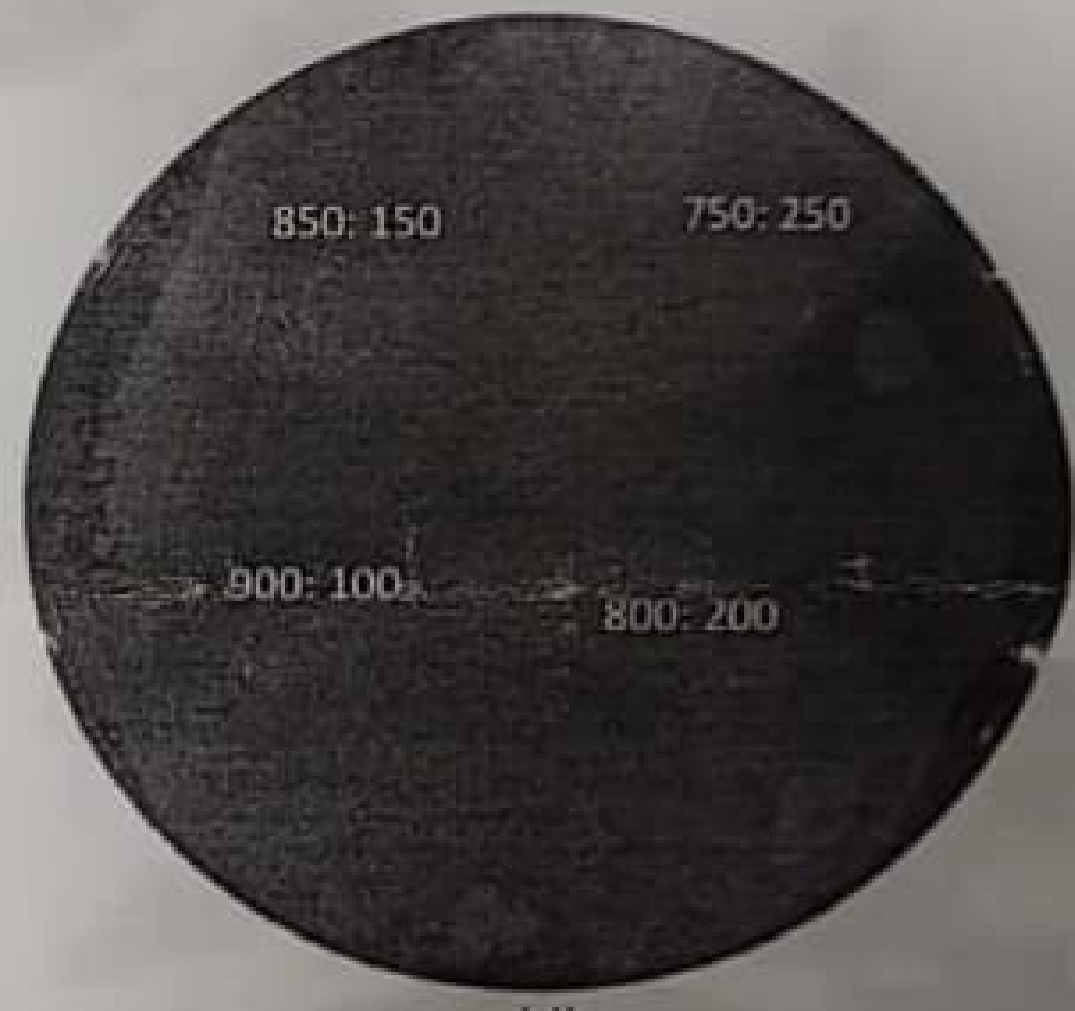

(d)

The above images shows the antimicrobial study report of the samples against the microbe, *C.albicans*. Figure (a) different ratios of Triphala kashayam (TK) : Gomutra Arkam Prepared (GAP) incubated for 24 hrs, figure (b) different ratios of Triphala kashayam (TK) : Gomutra Arkam Prepared (GAP) incubated for 36 hrs, figure (c) different ratios of Triphala kashayam (TK) : Gomutra Arkam market sample (GAM) incubated for 24 hrs, and figure (d) different ratios of Triphala kashayam (TK) : Gomutra Arkam market sample (GAM) incubated for 36 hrs.

Inference

Sample Name/ Ratios	900: 100	850:150	800:200	750:250
TK: GAP (24 hrs)	34mm	28mm	35mm	32mm
TK: GAP (36 hrs)	Nil	Nil	Nil	Nil
TK: GAM (24 hrs)	36mm	30mm	37mm	30mm
TK: GAM (36 hrs)	Nil	Nil	Nil	Nil

The antimicrobial zones of the samples in different incubation time.

After incubation of 24 hours the samples showed results whereas after incubation of 36 hours the results didn't persist due to over growth of the microbe.

Processed by: Minsha M G

Prepared by: Minsha. M.G

Verified by:

Research Coordinator, Laboratory

ACARA
